# Transcriptional factor ATF3 impairs KSHV lytic replication by suppressing the expression of viral bZIP protein K8

**DOI:** 10.1371/journal.ppat.1014222

**Published:** 2026-05-11

**Authors:** Jiazhen Dong, Xiaowei Liang, Yuncai Chen, Jiangwei Peng, Xiaoyi Sun, Jing Huang, Lei Bai, Ke Lan

**Affiliations:** 1 State Key Laboratory of Virology and Biosafety, College of Life Sciences, Wuhan University, Wuhan, China; 2 Frontier Science Center for Immunology and Metabolism, Wuhan University, Wuhan, China; 3 TaiKang Center for Life and Medical Sciences, Wuhan University, Wuhan, China; University of Southern California, UNITED STATES OF AMERICA

## Abstract

Kaposi’s Sarcoma-associated herpesvirus (KSHV) is a human gamma herpesvirus, establishing two different phases in its life cycle, latent infection and lytic replication. KSHV-encoded basic leucine zipper (bZIP) family of protein K8 (also called K-bZIP), an immediate-early protein, plays an indispensable role in KSHV lytic replication. Recombinant virus with K8 deletion exhibits an aberrant gene expression pattern and impairs virus production in KSHV lytic replication. However, the regulatory mechanisms of K8 itself expression have not been fully elucidated. In this study, we identified a host protein named activating transcription factor 3 (ATF3), a member of the CREB/ATF family of transcription factors, interacting with K8 in nucleus. Meanwhile, we further determined that the bZIP domain of ATF3 is necessary for their interaction. Besides, we demonstrated that ATF3 works as an antiviral factor in KSHV lytic replication, inhibiting viral genes expression and impairing the production of progeny virions. Mechanistically, ATF3 associates with K8, leading to the repression of *K8* promoter activity and thereby decreases the expression of K8 at both RNA and protein levels. Interestingly, we also verified that KSHV-encoded latency-associated nuclear antigen (LANA) could antagonize the antiviral activity of ATF3 by repressing *ATF3* promoter activity to reduce its expression. Collectively, we identified that the transcription factor ATF3 as a novel binding partner of K8 and their interaction represses *K8* promoter activity, leading to the reduction of K8 expression and consequently impairing KSHV lytic replication, which provide new insights into the development of novel antiviral strategies.

## Author summary

K8, also named K-bZIP, a viral encoded transcriptional factor, utilizes its bZIP domain to interact with various cellular and viral proteins, mainly working as a transcriptional factor to regulate gene transcriptional levels and playing indispensable roles in KSHV lytic replication. Here, we reported a host bZIP transcription factor named ATF3 as a novel binding protein of *K8* and demonstrated that their interaction represses *K8* promoter activity, leading to the reduction of K8 at both transcription and protein levels, which consequently impairs KSHV lytic replication. To evade the antagonism of ATF3, KSHV utilizes another transcriptional factor, LANA, to suppress *ATF3* promoter activity, leading to the reduction of ATF3 expression. These findings reveal a regulatory mechanism underlying K8 and host, further confirm the significance of K8 in KSHV lytic replication, and identified transcriptional factor ATF3 might be a promising therapeutic target for KS.

## Introduction

Kaposi’s sarcoma-associated herpesvirus (KSHV), also known as human herpesvirus 8 (HHV8), is a member of the gamma-herpesvirus family [1]. KSHV is a large double-stranded DNA virus with a genome of about 165 kb, which was first identified in an AIDS-associated Kaposi’s sarcoma (AIDS-KS) tissues, and has since been etiologically associated with several human diseases, including endothelial-derived Kaposi’s sarcoma, a B cell malignancy named as primary effusion lymphoma (PEL), a subset of multicentric Castleman’s disease (MCD), and KSHV-associated inflammatory cytokine syndrome (KICS) [2–5]. Similar with other herpesviruses, KSHV establishes two phases, latent infection and lytic replication, in its lifecycle. During the latent infection phase, the viral genome forms covalently closed circular episome tethered to the host chromosome and only some limited latent genes are expressed, but no virus particles are produced. Under specific physiological conditions, KSHV in latent infected cells can be induced to enter the lytic cycle, expressing extensive viral genes, producing infectious virions to infect new target cells, and leading to the death of host cells [6–8]. The precise and intricate regulation of viral latency and lytic replication maintains KSHV persistent infection and promotes tumorigenesis [9–11].

KSHV ORF K8 encodes an immediate-early protein that is activated by and expressed after KSHV RTA (replication and transcription activator) [12]. K8 is a 237-amino-acid protein, harboring two functional domains: a transcription activation domain at the N terminus (amino acids 1–121) and a prototypic basic region-leucine zipper (bZIP) domain at its C terminus (amino acids 122–237), and thus K8 also called K-bZIP [13]. Of note, K8 is a multifunctional protein in KSHV lytic replication, involved in several key processes such as regulation of various genes’ transcriptional expression, participation in the SUMOylation modification regulation, initiation of viral DNA replication, etc. [14–16]. For example, K8 alone was reported to be able to activate 21 KSHV promoters, including tegument protein ORF19, glycoprotein gH, capsid proteins ORF11 and ORF65, etc. [17]. In addition to its roles as a transcription activator, K8 also represses the transactivation of some RTA-dependent viral promoters, including ORF57 and K8 itself [18,19]. Meanwhile, K8 also interacts with cellular proteins such as CREB-binding protein (CBP), transcription factor p53 and cellular chromatin-remodeling factor hSNF5 to suppress their-mediated transcriptional activity [20,21]. In addition, K8 has been confirmed being a SUMO E3 ligase, catalyzing the SUMOylation of viral and host proteins and modulating the global SUMOylation level of cells, which plays an important role in KSHV lytic replication [22–24]. Moreover, K8 indirectly binds to the C/EBP sites of KSHV lytic origins of DNA replication (oriLyt), initiating the oriLyt-dependent DNA replication [25–27]. In a word, K8 has been reported that associates with various cellular and viral proteins to participate in regulating the lytic replication of KSHV, however, the regulatory and molecular mechanisms of K8 itself expression have not been fully elucidated.

Activating transcription factor 3 (ATF3), a nuclear protein with 181 amino acids, is ubiquitously expressed in mammalian tissues and belongs to the ATF/CREB family of transcription factors containing the basic leucine zipper (bZIP) domain [28,29]. ATF3 is a stress-responsive transcriptional factor, recognizes the canonical sequence 5’-TGACGTCA-3’ of a variety of promoters in the form of homodimers or heterodimers with other members of the ATF/CREB family, acting as a transcriptional activator or repressor to regulate its downstream target genes depending on the cell types and stimulus [30–40]. For example, in the response to cellular growth, ATF3 works as a transcriptional activator to up-regulate the expression of the anti-apoptotic genes, such as *HSP27*, *RGS1* etc., inhibiting cellular apoptosis [41,42]. Besides, ATF3 also can work as transcriptional repressor by binding to regulatory sites on the *Ifnb1* promoter to inhibit the production of pro-inflammatory cytokines to evade inflammatory response [35]. Moreover, ATF3 utilizes its bZIP domain, a well characterized mediator of protein-protein interaction, to associate with various cellular proteins, including p53 and Smad3, to regulate their functions and indirectly affect downstream transcription mediated by these interacted proteins [34,37,38,40]. ATF3 binds the C terminus of p53, impairing its ubiquitination and preventing p53 from MDM2-mediated degradation, leading to the increased transcription from p53-regulated promoters [40]. Furthermore, recent research has demonstrated that ATF3 inhibits viral replication while also can be hijacked by viruses to enhance viral replication, depending on the specific host-virus interaction environment, as well as the species of virus infected [43]. For example, ATF3 inhibits Zika virus (ZIKV) infection by promoting the transcriptional levels of specific innate immune genes such as *RIG-I*, *STAT1*, and *ISG15*, while simultaneously inhibiting autophagy-related genes including *Beclin-1* and *ATG5* to destroy autophagy, which eventually undermines ZIKV infection [44–46]. Interestingly, ATF3 has been confirmed to facilitate Herpes Simplex Virus 1 (HSV-1) to maintain the latent infection in ganglia by in synergy with STAT3 to block viral reactivation and cellular apoptosis [47,48]. Overall, ATF3 exhibits a different regulatory function in multiple biological processes, indicating its complicated and critical roles, however, research about the function and mechanism of ATF3 in KSHV lytic replication remains largely unclear.

Despite extensive studies about how K8 regulates cellular and viral gene expression, analyses of the regulatory mechanisms of K8 itself expression are lacking. In this study, ATF3, a transcriptional factor containing the bZIP domain, was identified as a potential K8-binding protein from the immunoprecipitation-based mass spectrometry analysis of both endogenous and exogenous K8 in KSHV-positive cells. We therefore firstly verified the interaction between K8 and ATF3 and further determined the bZIP domain of ATF3 is required for their interaction. We then demonstrated that ectopic expression of ATF3 obviously impairs viral lytic replication, whereas knockdown of ATF3 with siRNAs substantially enhanced viral lytic replication. Mechanistically, ATF3 associates with K8, leading to the repression of *K8* promoter activity and thereby impairing K8 expression at both RNA and protein levels. Interestingly, we also found that latency-associated nuclear antigen (LANA), a viral encoded transcriptional factor, could repress *ATF3* promoter activity in a dose-dependent manner, leading to the reduction of ATF3 expression, which antagonizes the antiviral roles of ATF3 in KSHV lytic replication. Taken together, these results reveal that the transcriptional factor ATF3 is a new host binding partner of KSHV K8, suppresses *K8* promoter activity, lead to the reduction of K8 expression and thereby impairs viral lytic replication, which provides new insights into the development of potential therapeutic target for KSHV-related malignances.

## Results

### KSHV bZIP protein K8 interacts with ATF3

**Fig 1:**
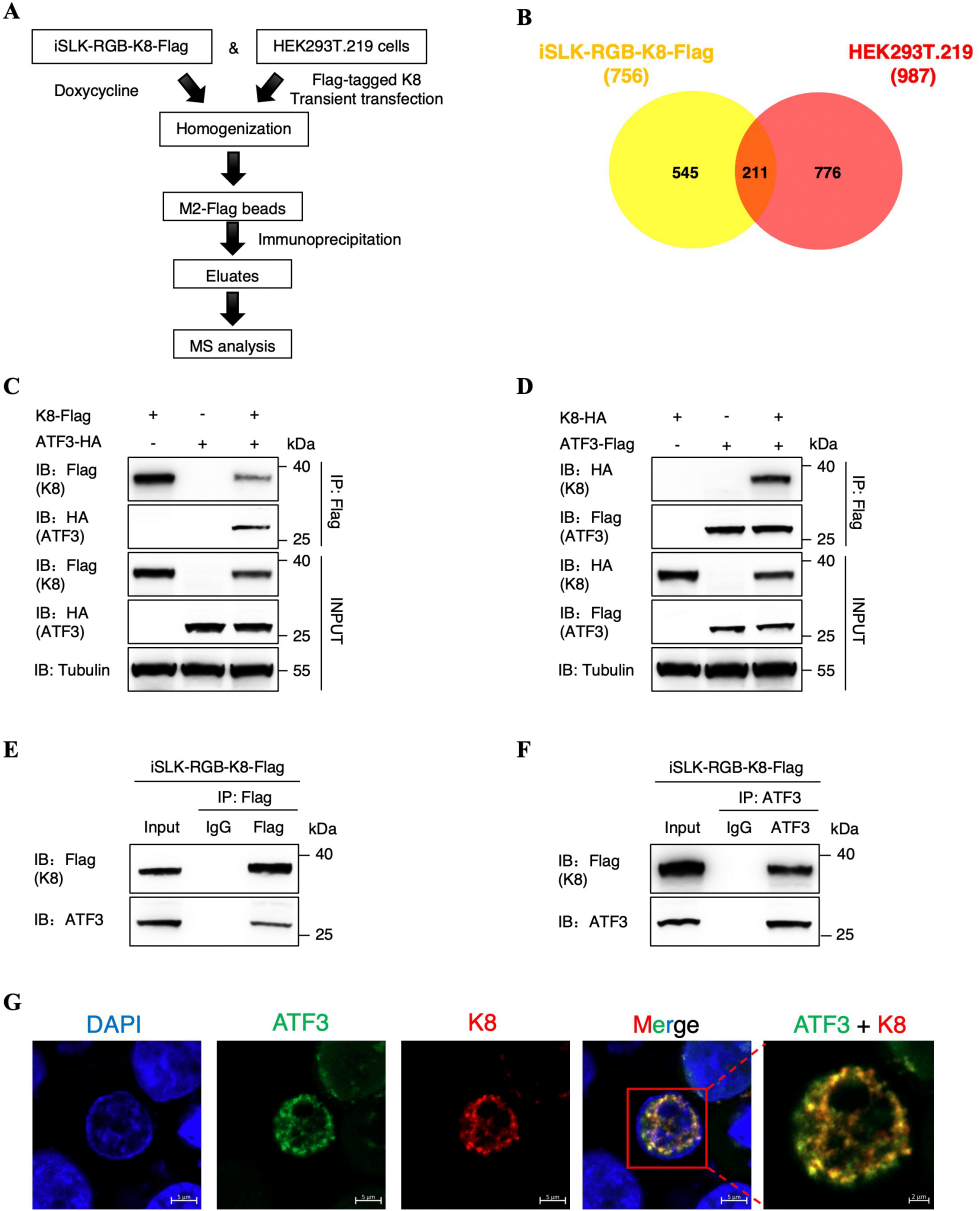
KSHV bZIP protein K8 interacts with ATF3. **(A)** Schematic strategy for purification and identification of K8 binding proteins with IP assay. iSLK-RGB-K8-Flag cells were induced by doxycycline (1 μg/ml) for 72 hours, while plasmid expressing Flag-tagged K8 was transient transfection into HEK293T.219 cells for 48 hours. Cell lysates were performed to affinity purification by immunoprecipitation with FLAG M2 beads. The purified elutes were boiled in SDS-PAGE loading buffer and then were analyzed by MS. **(B)** Venn diagram showing the overlaps of differentially candidate K8 binding proteins in iSLK-RGB-K8-Flag and HEK293T.219 cells. **(C)** HEK293T cells were transfected with K8-Flag alone, with ATF3-HA alone or with both K8-Flag and ATF3-HA. **(D)** HEK293T cells were transfected with ATF3-Flag alone, with K8-HA alone or with both ATF3-Flag and K8-HA. For C and D, cell lysates were immunoprecipitated with an anti-Flag antibody and were then analyzed by immunoblotting with the indicated antibodies. **(E and F)** Co-IP of endogenous K8 and ATF3 in iSLK-RGB-K8-Flag cells. Expression of K8 in the cells was induced by doxycycline (1 μg/ml) for 48 hours, and cell lysates were subjected to immunoprecipitation with the anti-Flag antibody or mouse IgG control antibody (E); the anti-ATF3 antibody or rabbit IgG control antibody (F). Purified proteins, along with input samples, were subjected to immunoblotting with the indicated antibodies. **(G)** Colocalization of ATF3 and K8 in HEK293T cells. Following transfection with ATF3-Flag and K8-HA, cells were fixed with 4% paraformaldehyde and then stained with mouse anti-Flag antibody and rabbit anti-HA antibody, followed by incubation with goat anti-mouse IgG conjugated with Alexa Fluor 488 and goat anti-rabbit IgG conjugated with Alexa Fluor 555 to visualize the stained ATF3 and K8 proteins, respectively. Nuclei were labelled with DAPI. Cells were analyzed by Zeiss confocal microscopy and representative images with scale bars were shown.

To further understand the regulatory mechanisms underlying K8 itself expression, we firstly utilized bacterial artificial chromosome (BAC) to construct a recombinant virus in which a Flag tag is fused to the C terminus of K8 in KSHV genome (S1A Fig) and established a stable cell line infected with this recombinant virus named iSLK-RGB-K8-Flag. Immunoblotting and qPCR experiments indicated the correct insertion of Flag sequence and lack of any alteration on KSHV lytic replication (S1B–S1D Fig). We then used the Flag-tag-based immunoprecipitation coupled with mass spectrometry to identify novel host factors interacting with K8 in iSLK-RGB-K8-Flag cells and HEK293T.219 cells after doxycycline induction and transient transfection (Fig 1A). Based on the data of mass spectrometry, we drew the Venn diagram identifying 211 K8-interacting proteins enriched in both iSLK-RGB-K8-Flag cells and HEK293T.219 cells (Fig 1B). These results are summarized in S1 Table, and a host protein named ATF3 was first rank in candidate proteins that can potentially interact with K8. We then adopted co-immunoprecipitation (Co-IP) assays to confirm the interaction between K8 and ATF3 in HEK293T cells. We transfected Flag-tagged K8 and HA-tagged ATF3 individually or together in cells, which demonstrated that ATF3 was coimmunoprecipitated with K8 (Fig 1C). Similarly, the reverse Co-IP experiment showed that the K8 protein was specifically coimmunoprecipitated with ATF3 (Fig 1D). Additionally, we also performed endogenous Co-IP assays in iSLK-RGB-K8-Flag cells after doxycycline induction. After immunoprecipitating with anti-Flag targeting K8 or anti-ATF3 antibody, endogenous ATF3 were observed to form a complex with K8 in iSLK-RGB-K8-Flag cells (Fig 1E and 1F). Moreover, to further confirm the results of immunoprecipitation, we performed an immunofluorescence assay (IFA) in HEK293T cells to determine whether ATF3 and K8 could be colocalized in the same cellular compartment. Flag-tagged ATF3 and HA-tagged K8 were co-transfected transiently in HEK293T cells. As expected, ATF3 and K8 were observed to be co-localized in the nuclear compartment (Fig 1G). Meanwhile, to further validate the physiological relevance between ATF3 and K8, we also performed endogenous IFA assay in KSHV-positive iSLK-BAC16 cells, which demonstrated that ATF3 and K8 also co-localized in the nucleus during viral lytic replication (S2 Fig). Collectively, these results confirm that the host ATF3 is a novel K8-interacting protein, and their interaction is in the nucleus.

### Mapping the interaction domains in K8 and ATF3

**Fig 2:**
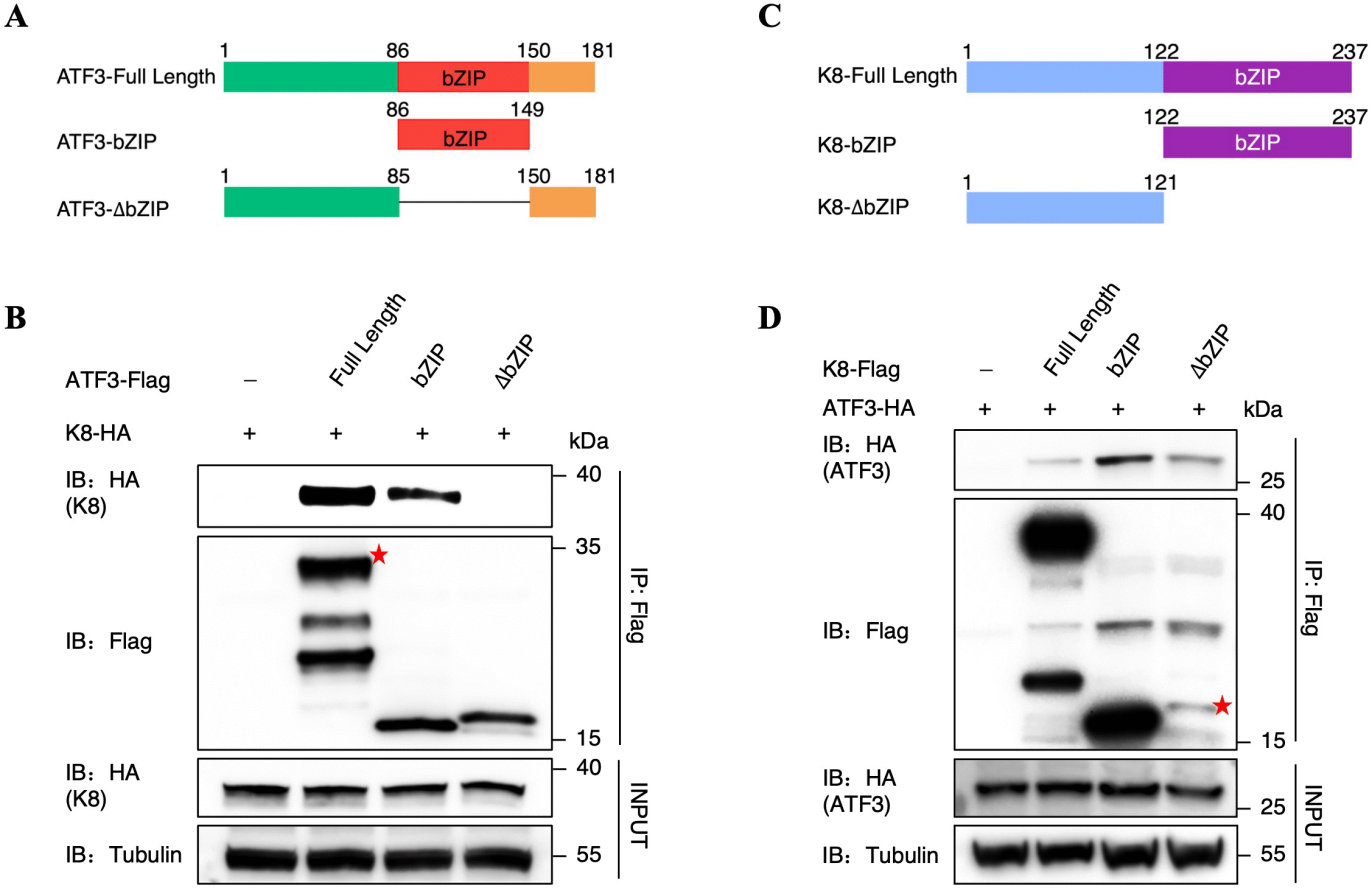
Mapping the interaction domains in K8 and ATF3. **(A)** Truncated versions of ATF3 are shown schematically, including ATF3-bZIP (86 aa-149 aa) and the bZIP region deletion truncation of ATF3 (ATF3-ΔbZIP). **(B)** Defining the K8-interacting domain of ATF3. Co-IP and immunoblotting of HEK293T cells transfected with HA-tagged K8 along with plasmids expressing the empty vector, Flag-tagged ATF3 truncations or the full-length ATF3. **(C)** Schematic diagram of the K8 protein and its truncations, including K8-bZIP (122 aa-237 aa) and the bZIP region deletion truncation of K8 (K8-ΔbZIP, 1 aa-121 aa). **(D)** Defining the ATF3-interacting domain of K8. Co-IP and immunoblotting of HEK293T cells transfected with HA-tagged ATF3 along with plasmids expressing the empty vector, Flag-tagged K8 truncations or the full-length K8.

ATF3, a 181-residue protein, has been shown to consist of major three well-defined domains, including an N-terminal domain (from 1 aa to 85 aa), a basic ZIP domain (from 86 aa to 149 aa) essential for protein-protein interaction, and a C-terminal activation domain (from 150 aa to 181 aa) [48–50]. To evaluate the importance of individual ATF3 regions responsible for the interaction with K8, we generated a series of Flag-tagged ATF3 truncations (Fig 2A). We then co-transfected K8 and full-length ATF3 or ATF3 truncations into HEK293T cells. The Co-IP assay showed that the bZIP domain (from 86 aa to 149 aa) is the crucial region responsible for the association of ATF3 and K8 (Fig 2B). Meanwhile, a similar approach was employed to determine the minimum region of K8 required for its interaction with ATF3. A series of Flag-tagged K8 truncation mutants were generated (Fig 2C) [14], and the ability of these mutants to interact with ATF3 was assessed. Interestingly, as shown in Fig 2D, the full-length of K8 and its truncation mutants all interact with ATF3. Taken together, these results suggested that the interaction between ATF3 and K8 depends on the bZIP domain of ATF3.

### Ectopic expression of ATF3 impairs KSHV lytic replication

**Fig 3:**
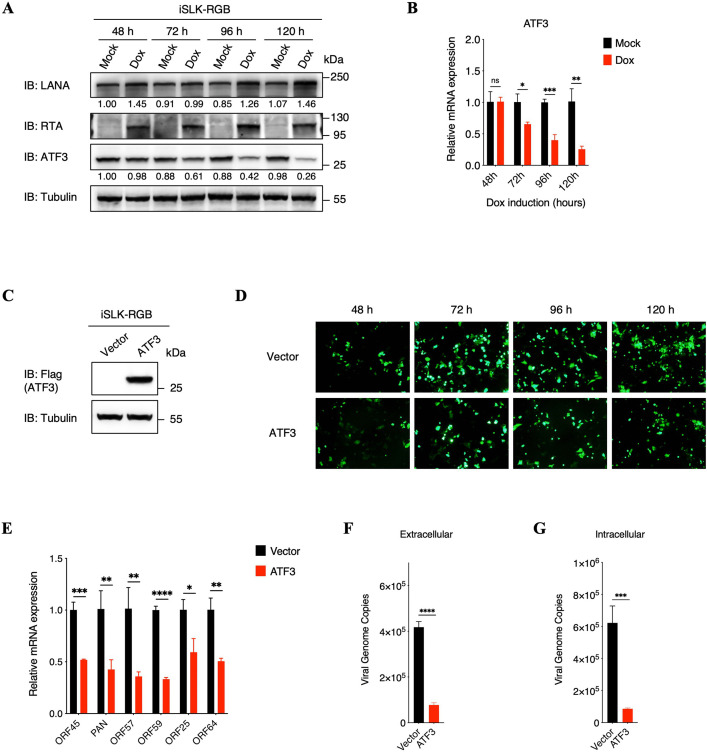
Ectopic expression of ATF3 impairs KSHV lytic replication. **(A and B)** iSLK-RGB cells were treated with or without doxycycline (1 μg/ml) at different time points as indicated. The expression kinetics of LANA, RTA and ATF3 were detected by immunoblotting (A) and the mRNA expression of ATF3 was determined by qPCR analysis (B). The protein level of LANA and ATF3 were quantified by densitometry and normalized to Tubulin level. **(C)** iSLK-RGB cells were stably transfected with lentiviruses containing an empty vector plasmid or a Flag-tagged ATF3 expression plasmid, named iSLK-RGB-Vector and iSLK-RGB-ATF3, respectively. The overexpression of ATF3 was detected by immunoblotting. **(D)** iSLK-RGB-Vector and iSLK-RGB-ATF3 cells were treated with doxycycline (1 μg/ml) at different time points as indicated. Fluorescence microscopy images of eGFP-positive cells among iSLK-RGB-Vector and iSLK-RGB-ATF3 cells were shown. **(E to G)** iSLK-RGB-Vector and iSLK-RGB-ATF3 cells were induced by doxycycline (1 μg/ml) for 72 hours, and total RNA was extracted from cells to investigate the transcriptional level of indicated genes (E); the extracellular virion DNA (F) and the intracellular viral genomic DNA (G) were extracted from cell supernatants or cell lysates to quantify the KSHV genomic DNA copy numbers by qPCR analysis. For B and E to G, bars represent means ±SEM of triplicates from three independent experiments. The P values were calculated using Student’s t-test (two sides). *P < 0.05, **P < 0.01, ***P < 0.001, ****P < 0.0001, ns indicates no significance.

Subsequently, we further characterized the roles of ATF3 in KSHV lytic replication. We firstly detected the kinetics of ATF3 expression in iSLK-RGB cells at different time points after doxycycline induction, which demonstrated that both protein and RNA level of ATF3 were suppressed, especially at the latter time points of viral lytic replication (Fig 3A and 3B). Interestingly, by analyzing the public KSHV RNA-seq datasets [51,52], we found that ATF3 expression was also markedly inhibited in KSHV-positive PEL cells, including BCBL-1 and BC-3, compared with KSHV-negative PEL cells such as BJAB, DG75 and Raji (S3A and S3B Fig). Similar with these findings, an obvious reduction in ATF3 protein expression was consistently observed in KSHV-positive BJAB-KSHV and KMM cells, comparing with KSHV-negative BJAB and MM cells (S3C and S3D Fig). These results collectively suggested the potential anti-viral capacity of ATF3 in KSHV infection.

Therefore, to further evaluate the roles of ATF3, we constructed an iSLK-RGB-ATF3 cell line that stably expressed Flag-tagged ATF3 in KSHV lytic replication (Fig 3C). Considering iSLK-RGB cell line that was stably infected with a reporter KSHV virus named red-green-blue-BAC16, which contained three fluorescent protein expression cassettes, including EF1α promoter-red fluorescent protein (RFP), PAN promoter-enhanced green fluorescent protein (eGFP) and K8.1 promoter-blue fluorescent protein [53], we firstly adopted fluorescence microscopy to evaluate the roles of ATF3 in KSHV lytic replication, which demonstrated that the number of eGFP-positive cells was greatly reduced after ATF3 ectopic expression compared with control cells (Fig 3D). Besides, we detected the transcriptional levels of various KSHV genes by real-time qPCR, which showed that the immediate early, early and late genes of viral lytic genes were all obviously inhibited in ATF3 stable expression cells (Fig 3E). Meanwhile, comparing with control cells, both extracellular and intracellular KSHV virions were obviously reduced in ATF3 overexpressed cells (Fig 3F and 3G).

Additionally, in keeping with the results of ATF3 overexpression, treatment of iSLK-RGB cells with the ATF3-specific agonist, ATF3 inducer 1 [54–57], also effectively restricted KSHV lytic replication, as evidenced by an obvious reduction in the number of eGFP-positive cells, viral gene expression and KSHV virions production (S4A–S4D Fig). However, in contrast to the full-length ATF3, neither of the two ATF3 truncation mutants exhibited a significant inhibitory effect on KSHV lytic replication (S5A–S5C Fig), indicating that their ability to repress viral lytic replication was largely abrogated. Taken together, these results demonstrated that ectopic expression of full-length ATF3 efficiently impairs KSHV lytic replication.

### Downregulation of endogenous ATF3 enhances KSHV lytic replication

**Fig 4:**
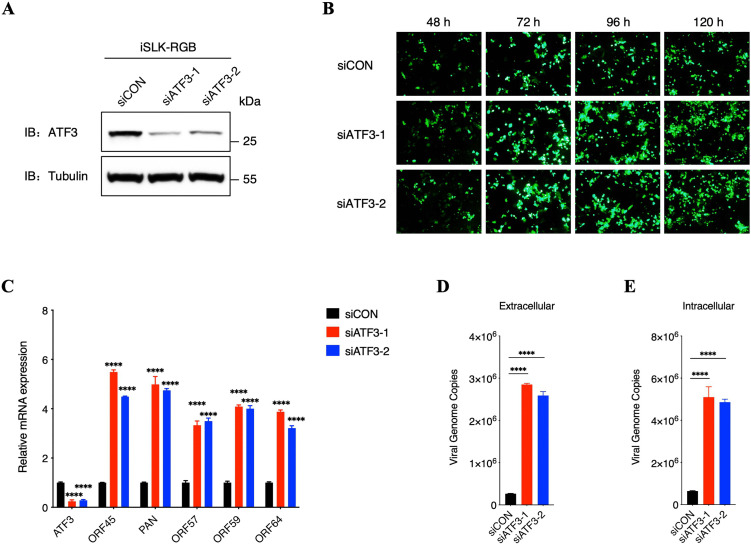
Downregulation of endogenous ATF3 enhances KSHV lytic replication. **(A)** iSLK-RGB cells were transfected with control siRNA and two ATF3-specific siRNAs. The knockdown efficiency was determined by immunoblotting. **(B)** At 6 hours after transfection, cells were induced by doxycycline (1 μg/ml) at different time points as indicated. Fluorescence microscopy images of eGFP-positive cells were shown. **(C to E)** At 6 hours after transfection, cells were induced by doxycycline (1 μg/ml) for another 72 hours. The ATF3 and KSHV gene transcription level were analyzed by qPCR (C); the extracellular virion DNA (D) and the intracellular viral genomic DNA (E) were extracted from cell supernatants or cell lysates to quantify the KSHV genomic DNA copy numbers by qPCR analysis. For C to E, bars represent means ±SEM of triplicates from three independent experiments. The P values were calculated using Student’s t-test (two sides). ****P < 0.0001.

To further confirm the effect of ATF3 on KSHV lytic replication, we transfected iSLK-RGB cells with two ATF3-specific siRNAs or equal amounts of control siRNA 24 h before induction with doxycycline (Fig 4A). Subsequently, we detected the ratio of cells expressed eGFP, the transcriptional levels of various lytic genes including immediate early, early and late genes, as well as both extracellular and intracellular KSHV virions. As expected, knockdown of endogenous ATF3 expression in iSLK-RGB cells obviously enhanced the number of cells expressed eGFP (Fig 4B), the transcription of lytic genes (Fig 4C), and virion production (Fig 4D and 4E) compared to those in cells transfected with the scrambled siRNA control. To strengthen these conclusions, we used another KSHV-positive cell line, BCBL-1. We treated cells with tetradecanoyl phorbol acetate (TPA) following the inhibition of ATF3 expression with siRNAs. Consistent with the results of iSLK-RGB, the transcriptional levels of a variety of lytic genes as well as the production of progeny viruses were substantially increased in BCBL-1 with siRNAs (S6A–S6D Fig). Collectively, these results demonstrated that ATF3 plays an antiviral role in KSHV lytic replication.

### ATF3 associates with K8 to repress *K8* promoter activity

**Fig 5:**
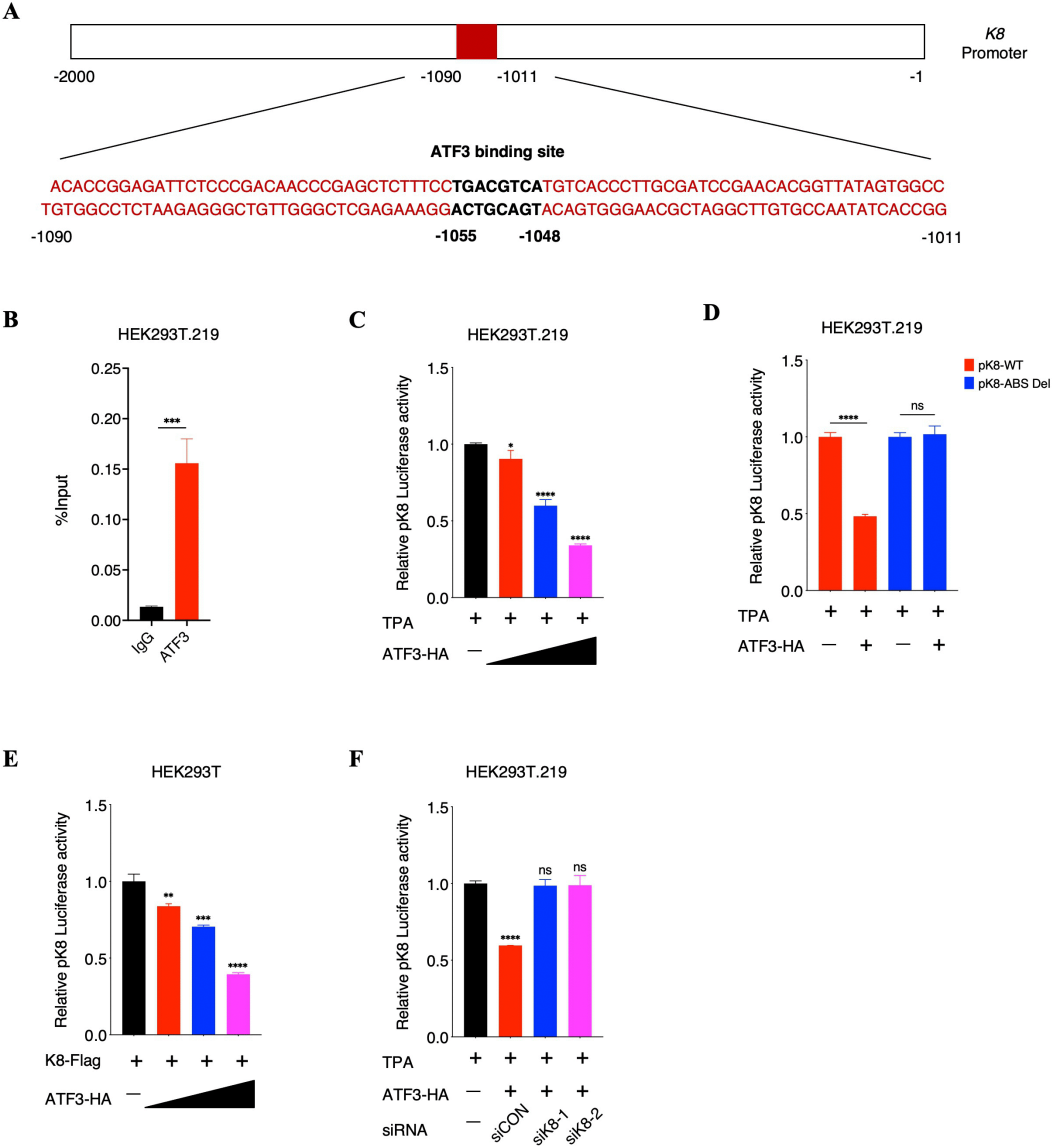
ATF3 associates with K8 to repress *K8* promoter activity. **(A)** Schematic diagram of *K8* promoter region (from -2000 bp to -1 bp). The ATF3 binding site (from -1055 bp to -1048 bp) is marked. **(B)** Validation of ATF3 binding at the *K8* promoter. HEK293T.219 cells were induced by TPA (20 ng/ml) for 48 hours and were subjected to ChIP assays using immunoglobulin G or the ATF3 antibody. The amounts of precipitated DNA were quantified by qPCR using primers amplifying the regions containing ATF3 binding site. **(C)** ATF3 suppresses *K8* promoter activity in a dose-dependent manner in HEK293T.219 cells. HEK293T.219 cells were transfected with pK8 dual-reporter plasmid and increasing amounts of ATF3-expressing plasmids. At 6 hours after transfection, cells were induced by TPA (20 ng/ml) for another 48 hours and cells were harvested and lysed in lysis buffer to detect luciferase activity. **(D)** ATF3 has no effect on the activity of *K8* promoter with deletion of ATF3 binding sites (pK8-ABS Del) in HEK293T.219 cells. HEK293T.219 cells were transfected with pK8-WT or pK8-ABS Del dual-reporter plasmids and ATF3-expressing plasmids. At 6 hours after transfection, cells were induced by TPA (20 ng/ml) for another 48 hours and cells were harvested and lysed in lysis buffer to detect luciferase activity. **(E)** With K8 overexpression, ATF3 suppresses *K8* promoter activity in a dose-dependent manner in HEK293T cells. HEK293T cells were transfected with pK8 dual-reporter plasmid, constant amounts of K8-expressing plasmids and increasing amounts of ATF3-expressing plasmids. At 48 hours after transfection, the cells were harvested and lysed in lysis buffer to detect luciferase activity. **(F)** ATF3 has no effect on *K8* promoter activity when K8 expression is suppressed with siRNAs in HEK293T.219 cells. HEK293T.219 cells were transfected with control siRNA and two K8-specific siRNAs. At 24 hours after siRNAs transfection, cells were transfected with pK8 dual-reporter plasmids and ATF3-expressing plasmids. At 6 hours after the plasmids-transfection, cells were induced by TPA (20 ng/ml) for another 48 hours and cells were harvested and lysed in lysis buffer to detect luciferase activity. For B to F, bars represent means ±SEM of triplicates from three independent experiments. The P values were calculated using Student’s t-test (two sides). *P < 0.05, **P < 0.01, ***P < 0.001, ****P < 0.0001, ns indicates no significance.

Subsequently, we further discussed the mechanisms underlying how ATF3 impairs KSHV lytic replication. Since ATF3 can recognize the canonical sequence 5’-TGACGTCA-3’ in the form of homodimers or heterodimers to function as a transcriptional activator or repressor to regulate the target gene expression [28,29], we thus analyzed the sequences of the whole KSHV genome and identified the ATF3-binding site (from -1055 bp to -1048 bp) in the *K8* promoter (Fig 5A), suggesting that ATF3 is a transcriptional target of K8. To identify the link between ATF3 and K8, we firstly conducted ChIP-qPCR assays in HEK293T.219 cells after the induction of TPA. As expected, the ATF3 binding elements immunoprecipitated by the ATF3 antibody in the *K8* promoter were significantly enriched compared with the control IgG antibody (Fig 5B). We then cloned the DNA fragment of the *K8* promoter region (from -2000 bp to -1 bp) into the pGL3-Enhancer luciferase reporter vector and performed luciferase reporter assays. Interestingly, ATF3 overexpression had completely no effect on *K8* promoter in HEK293T cells (S7A Fig), but repressed *K8* promoter activity in a dose-dependent manner after the induction of TPA in HEK293T.219 cells (Fig 5C). Meanwhile, ATF3 overexpression hardly affect the activity of *K8* promoter with ATF3-binding site deletion (pK8-ABS Del) in HEK293T.219 cells (Fig 5D). These results collectively suggested that ATF3 utilizes viral proteins to corporately suppress *K8* promoter activity. Considering the interaction between ATF3 and K8, we thus speculated that it is interaction between ATF3 and K8 that results in the repression of *K8* promoter activity. To verify our speculation, we co-transfected the increasing amounts of ATF3 with a constant amount of K8 plasmids into HEK293T cells and performed luciferase reporter assays on *K8* promoter. As expected, in the presence of K8 protein expression, ATF3 repressed *K8* promoter activity in a dose-dependent manner (Fig 5E). Meanwhile, we demonstrated that when K8 expression was inhibited by siRNAs, the repression of ATF3 on *K8* promoter was greatly abolished in HEK293T.219 cells (Fig 5F). Moreover, to further substantiate our hypothesis, we detected the effect of full-length ATF3 and ATF3 bZIP domain deletion truncation on the repression of *K8* promoter in HEK293T.219 cells. We demonstrated that comparing with full-length ATF3, ATF3 bZIP domain deletion truncation, which cannot bind to K8, was completely deprived of capacity to suppress *K8* promoter activity (S7B and S7C Fig). In conclusions, these results indicated that ATF3 can repress *K8* promoter activity, which requires the interaction with K8.

### ATF3 reducing K8 expression depends on its bZIP domain

**Fig 6:**
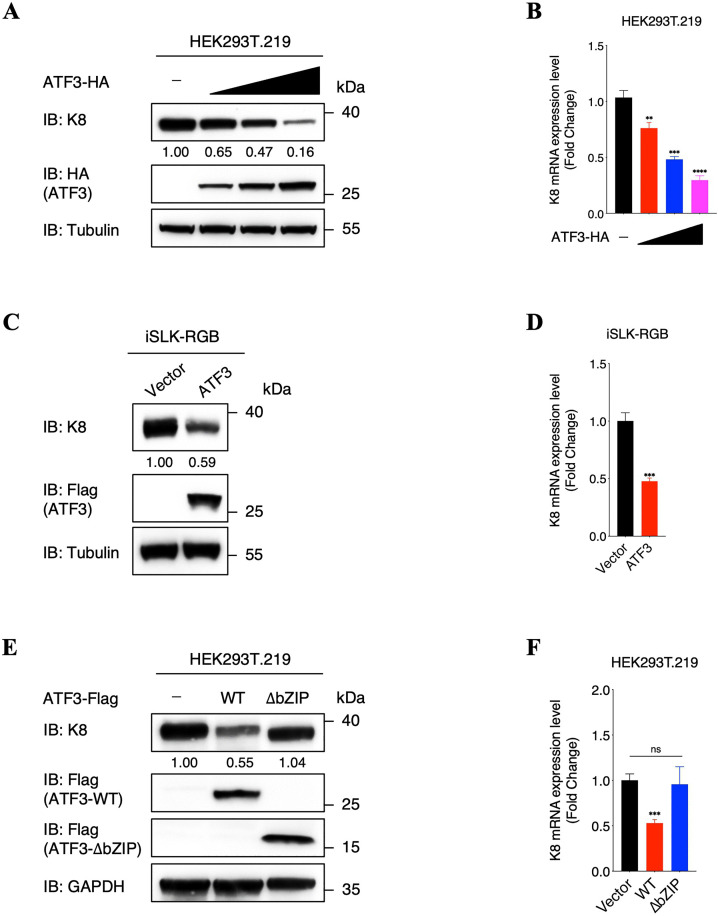
ATF3 reducing K8 expression depends on its bZIP domain. **(A and B)** Effect of ATF3 on endogenous K8 expression. HEK293T.219 cells were transfected with increasing amounts of ATF3-expressing plasmids. At 6 hours after transfection, cells were induced by TPA (20 ng/ml) for another 48 hours and cells were harvested and analyzed by immunoblotting with the indicated antibodies. The protein level of K8 was quantified by densitometry and normalized to Tubulin level (A); total RNA was extracted from cells to investigate the transcriptional level of K8 (B). **(C and D)** Effect of ATF3 on endogenous K8 expression. iSLK-RGB-Vector and iSLK-RGB-ATF3 cells were induced by doxycycline (1 μg/ml) for 72 hours and cells were harvested and analyzed by immunoblotting with the indicated antibodies. The protein level of K8 was quantified by densitometry and normalized to Tubulin level (C); total RNA was extracted from cells to investigate the transcriptional level of K8 (D). **(E and F)** Effect of wildtype ATF3 and bZIP deletion truncated ATF3 on endogenous K8 expression. HEK293T.219 cells were transfected with wildtype or bZIP deletion ATF3-expressing plasmids. At 6 hours after transfection, cells were induced by TPA (20 ng/ml) for another 48 hours and cells were harvested and analyzed by immunoblotting with the indicated antibodies. The protein level of K8 was quantified by densitometry and normalized to GAPDH level (E); total RNA was extracted from cells to investigate the transcriptional level of K8 (F). For B, D, F, bars represent means ±SEM of triplicates from three independent experiments. The P values were calculated using Student’s t-test (two sides). *P < 0.05, **P < 0.01, ***P < 0.001, ****P < 0.0001, ns indicates no significance.

Furthermore, to elucidate the biological significance of ATF3 repressing *K8* promoter, we firstly assess whether ATF3 have impacts on K8 expression. We transiently transfected increasing amounts of ATF3 in HEK293T.219 cells and examined the effect of ATF3 on endogenous K8 expression following TPA treatment, which showed that ATF3 decreased both protein and RNA levels of K8 in a dose-dependent manner (Fig 6A and 6B). Besides, we also detected the effect of ATF3 on K8 expression in another KSHV-positive iSLK-RGB cells after doxycycline induction. As expected, significant decreases in both K8 protein and RNA levels were observed in iSLK-RGB-ATF3 cells (Fig 6C and 6D). To further confirm our findings, we detected endogenous K8 expression after inhibiting ATF3 expression with siRNAs in both HEK293T.219 and iSLK-RGB cells. Consistently, we observed an obvious increase in both K8 protein and RNA levels in ATF3-knockdown cells compared to wild-type cells (S8A–S8D Fig). Moreover, we also demonstrated that the ATF3 bZIP domain deletion truncation, which cannot bind to K8, was completely deprived of capacity to reduce neither protein nor RNA levels of K8 in HEK293T.219 cells (Fig 6E and 6F). Collectively, these results indicated that ATF3 reduces K8 expression at both protein and RNA levels, which depends on the bZIP domain of ATF3.

### LANA decreases ATF3 expression by repressing its promoter

**Fig 7:**
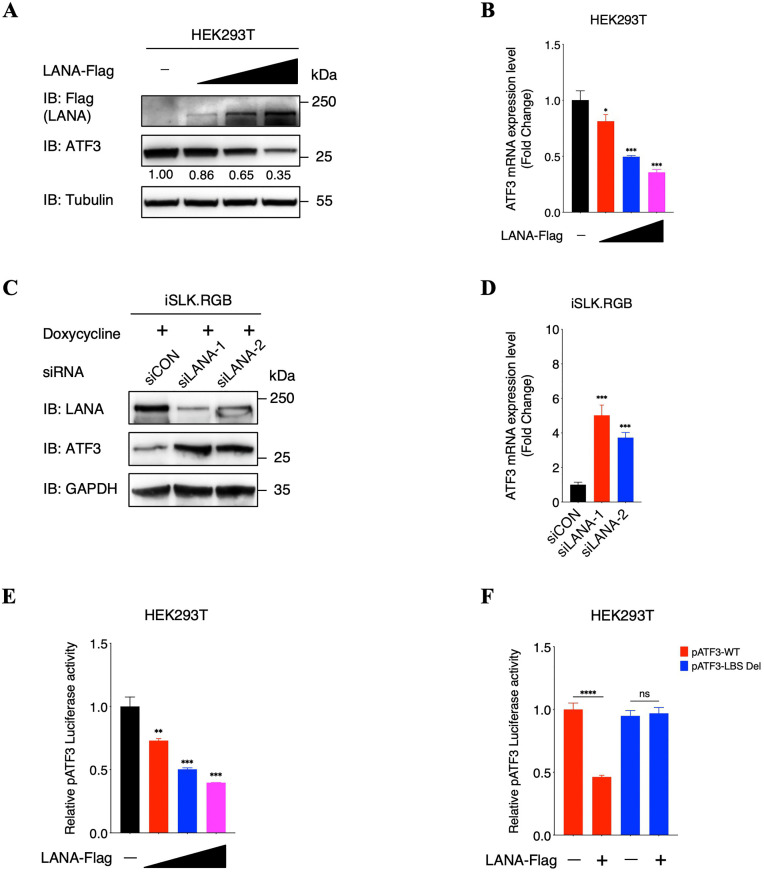
LANA decreases ATF3 expression by repressing its promoter. **(A and B)** Effect of LANA on endogenous ATF3 expression. HEK293T cells were transfected with increasing amounts of LANA-expressing plasmids. At 48 hours after transfection, cells were harvested and analyzed by immunoblotting with the indicated antibodies. The protein level of ATF3 was quantified by densitometry and normalized to Tubulin level (A); total RNA was extracted from cells to investigate the transcriptional level of ATF3 (B). **(C and D)** Effect of LANA on endogenous ATF3 expression. iSLK-RGB cells were transfected with control siRNA and two LANA-specific siRNAs. At 6 hours after transfection, cells were induced by doxycycline (1 μg/ml) for another 72 hours and cells were harvested and analyzed by immunoblotting with the indicated antibodies (C); total RNA was extracted from cells to investigate the transcriptional level of ATF3 (D). **(E)** LANA suppresses *ATF3* promoter activity in a dose-dependent manner in HEK293T cells. HEK293T cells were transfected with pATF3 dual-reporter plasmid and increasing amounts of LANA-expressing plasmids. At 48 hours after transfection, cells were harvested and lysed in lysis buffer to detect luciferase activity. **(F)** LANA has no effect on the activity of *ATF3* promoter with deletion of LANA binding sites (pATF3-LBS Del) in HEK293T cells. HEK293T cells were transfected with pATF3-WT or pATF3-LBS Del dual-reporter plasmids and LANA-expressing plasmids. At 48 hours after transfection, cells were harvested and lysed in lysis buffer to detect luciferase activity. For B and D to F, bars represent means ±SEM of triplicates from three independent experiments. The P values were calculated using Student’s t-test (two sides). *P < 0.05, **P < 0.01, ***P < 0.001, ****P < 0.0001, ns indicates no significance.

We have demonstrated that ATF3 associates with K8 to repress *K8* promoter activity, leading to the reduction of K8 on both RNA and protein levels and thereby plays an antiviral role in KSHV lytic replication. Interestingly, ATF3 expression was suppressed during viral lytic replication (Fig 2A and 2B), we therefore speculated that KSHV has evolved some mechanisms to evade the antagonism of ATF3. Of note, we identified several putative LANA-binding sites by searching the promoter region of the *ATF3* gene (S9A Fig). Considering LANA expression was increased during KSHV lytic replication and it has been proved to work as a transcriptional factor to regulate gene expression [7,58–60], we thus put forward a hypothesis that KSHV-encoded LANA decreases ATF3 expression through repressing the activity of *ATF3* promoter, which antagonizes the antiviral activity of ATF3. To confirm our speculation, we firstly performed dose-dependent assays to detect the effect of LANA on endogenous ATF3 expression in HEK293T cells, which showed that LANA decreases both protein and RNA levels of ATF3 in a dose-dependent manner (Fig 7A and 7B). Besides, we also detected ATF3 expression in iSLK-RGB and HEK293T.219 cells after suppressing LANA expression with siRNAs, which demonstrated that ATF3 expression was obviously enhanced when LANA expression was inhibited (Figs 7C, 7D, S9B and S9C). To further determine our hypothesis that LANA inhibits ATF3 expression by repressing the *ATF3* promoter, we firstly performed ChIP-qPCR assays in HEK293T cells after the transient transfection of Flag-tagged LANA. As expected, the LANA binding sites immunoprecipitated by the Flag antibody in the *ATF3* promoter were markedly enriched compared with the control IgG antibody (S9D Fig). Subsequently, the DNA fragment of the *ATF3* promoter (from -1500 bp to -1 bp) was cloned into a luciferase reporter vector and co-transfected into HEK293T cells with the LANA-expressing plasmids. As shown in Fig 7E, with increasing amounts of LANA, the activity of the *ATF3* promoter was suppressed in a dose-dependent manner. Moreover, deleting all putative LANA-binding sites in the *ATF3* promoter largely abrogated the repression by LANA (Fig 7F). Collectively, these results indicated that LANA decreases ATF3 expression by repressing the activity of *ATF3* promoter.

## Discussion

**Fig 8:**
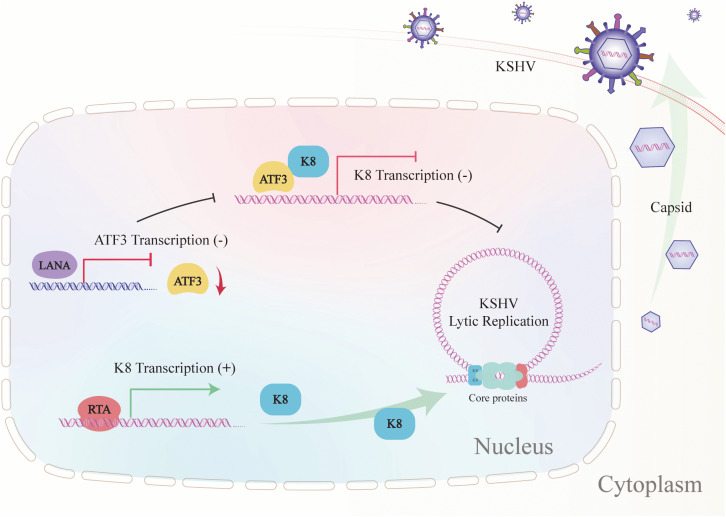
Working model for the roles of ATF3 in regulating KSHV bZIP protein K8. The cellular bZIP transcriptional factor ATF3 interacted with KSHV bZIP protein K8, leading to the repression of *K8* promoter activity, which obviously decreased K8 both transcriptional and protein expression and thereby greatly impairing KSHV lytic replication. In turn, KSHV encoded transcriptional factor LANA decreased ATF3 expression through repressing its promoter, which counteracts the antiviral activity of ATF3 and ensures a complete lytic replication of KSHV.

In this study, we identified a new binding-partner of KSHV-encoded bZIP protein K8, which is host bZIP transcriptional factor ATF3, and demonstrated that their interaction depends on the bZIP domain of ATF3. During lytic replication, ATF3 works as an antiviral role, decreasing viral gene expression and the production of viral progeny. Mechanistically, ATF3 associates with *K8* to repress *K8* promoter activity, leading to the reduction of K8 expression at both transcription and protein levels and thereby impairing KSHV lytic replication. Interestingly, to evade the antagonism of ATF3, KSHV utilizes viral transcriptional factor LANA to decrease the ATF3 expression through suppressing the activity of *ATF3* promoter, ensuring a complete lytic replication. In summary, our data establish a working model illustrating the roles of ATF3 in the regulation of K8 expression and KSHV lytic replication (Fig 8).

Moreover, by analyzing the sequences of the whole genome of KSHV, we identified two other ATF3-binding sites in KSHV, which were respectively in the *ORF7* promoter (from -1511 bp to -1504 bp) and the *ORF26* promoter (from -2366 bp to -2359 bp) (S10A and S10B Fig). We therefore speculated that ATF3 also participates in regulating these two genes’ promoter activity. To confirm our speculation, we firstly detected the effect of ATF3 on these two promoters with or without K8 expression in HEK293T cells. Consistent with results of *K8* promoter, ATF3 also suppressed the activity of both *ORF7* and *ORF26* promoters with association of K8 (S10C and S10D Fig). Subsequently, to further validate whether ATF3 regulates lytic genes primarily acts through K8, we firstly utilized BAC technology to construct a recombinant KSHV in which three stop codons were inserted into the N terminus of K8 and established a stable cell line that stably expressed this recombinant virus, named iSLK-RGB-K8-Stop. We then stably expressed Flag-tagged ATF3 in iSLK-RGB-K8-Stop and detected the transcriptional levels of lytic genes during KSHV lytic replication. We demonstrated that comparing with iSLK-RGB-WT cells, the transcriptional levels of lytic genes were obviously reduced in both iSLK-RGB-ATF3 and iSLK-RGB-K8-Stop cells during KSHV lytic replication. Of note, the inhibitory effect of ATF3 on lytic genes was greatly abrogated in cells where K8 expression was disrupted by BAC technology (S10E Fig), indicating that ATF3 employs a K8-dependent mechanism to negatively regulate viral lytic genes. Overall, our findings firstly elucidate the antiviral physiological functions of ATF3 in KSHV lytic replication and identify the diversity of targets of ATF3, which suggest that ATF3 probably serve as a potential therapeutic target for the prevention and treatment of several malignances induced by KSHV infection.

In addition, it has been confirmed that in the absence of K8 expression, viral DNA replication and virion production were severely impaired in both PEL cells and cells containing a K8 deletion BACmid [61,62], indicating the indispensable of K8 in KSHV lytic replication. However, current research about K8 mainly focuses on its function in KSHV lifecycle, working as a transcriptional factor or a SUMO E3 ligase [13,16–24,63]. Although it has been reported that K8 exists three post-translational modifications (PTMs), including phosphorylation, SUMOylation and acetylation, but these studies merely emphasized the impacts of these PTMs on the functions of K8 [14,22,64–67]. The regulatory mechanisms underlying K8 itself expression, however, remains largely elusive, only *Liao* et al., and colleagues have demonstrated that RTA activates K8 expression and K8 in turn interacts with RTA to repress RTA-mediated transactivation on K8 [18,19]. Therefore, to further explore the regulatory mechanism of K8 itself, we performed immunoprecipitation-based mass spectrometry of both endogenous and exogenous K8 in KSHV-positive cells. We for the first time demonstrated that K8 expression is regulated not only by viral-encoded proteins (KSHV RTA) but also by the host proteins (bZIP transcriptional factor ATF3). The study filled an important gap in our understanding of how K8 expression itself is regulated by host factors. Moreover, considering that K8 is a SUMO E3 ligase with specificity toward SUMO2/3 [22,24,63]. We also tested whether the binding with K8 is one of the reasons for the decreased expression level of ATF3 during KSHV lytic replication. We constructed a K8 SUMO E3 ligase dead mutant plasmid, K8-L75A-Flag, and evaluate its roles in HEK293T cells, which showed that neither wildtype nor mutant had an effect on endogenous ATF3 expression (S11A Fig). Moreover, we also utilized BAC technology to construct a recombinant KSHV containing the K8 SUMO E3 ligase dead mutation and establish the corresponding stable cells, named iSLK-RGB-K8-L75A-Flag. Consistent with the findings in HEK293T cells, ATF3 expression was inhibited in both wildtype and mutant cells during KSHV lytic replication (S11B Fig). These results collectively excluded the possibility that the downregulation of ATF3 expression was mediated by the SUMO E3 ligase activity of K8.

Furthermore, host stress responses, such as oxidative stress, DNA damage response, endoplasmic reticulum stress and etc., have been proved to serve as critical regulator that promote the switch of herpesviruses from latency to lytic replication [68–71]. For example, hypoxia-induced factors, XBP-1 and HIF-1α, promote the lytic reactivation of KSHV and Epstein-Barr virus (EBV) in latently infected cells through transactivating their respective lytic switch gene, RTA and BZLF1 [70,72,73]. Similarly, activation of ataxia telangiectasia mutated (ATM) protein kinase, a centered factor of DNA damage response (DDR), also was proved to promote both KSHV and human cytomegalovirus (HCMV) lytic replication [74–76]. Interestingly, previous studies have demonstrated the mutual interaction and regulation between ATF3 and cellular stress responses [77–79]. For instance, ATF3 modulates various oxidative stress related genes, NOX4 and Nrf2, to affect the several biological processes such as cell cycle, apoptosis, and drug resistance of cancer cells [80,81]. Likewise, DNA damage strongly induces ATF3 expression, while ATF3 in turn manipulates DNA damage signaling through regulating several related key factors, such as p53 and ATM [82,83]. Our studies for the first time reveal a regulatory circuit of ATF3-K8-LANA in controlling KSHV lytic reactivation, broaden our understanding of KSHV biology and provided a potential regulatory mechanism linking host stress pathway to herpesvirus reactivation.

More importantly, by analyzing the sequences of the whole genomes of human herpesviruses, including human herpesvirus-1 (HHV-1) to HHV-8, we interestingly found out that all human herpesviruses, except human herpesvirus 3, also named Varicella-zoster virus (VZV), harbor one or more ATF3-binding sites (S12 Fig), suggesting that ATF3 possibly participates in the regulation of the whole human herpesvirus family. Indeed, it has been reported that ATF3 enhances the accumulation of a long noncoding RNA (a latency-associated transcript, LAT) encoded by HSV-1 and together with LAT maintain the viral latent state in ganglia, playing a pro-viral function [48]. Besides, EBV also has been confirmed that it can hijack ATF3 to inhibit cellular apoptosis, accelerating growth, which lead to the promotion of gastric cancer tumorigenesis induced by EBV [84]. However, the detailed molecular mechanisms and biological significance of ATF3 in human herpesviruses remain largely elusive, which require further in-depth investigation and discussion. Collectively, these findings highlight the complexity and diversity of ATF3 roles in human herpesviruses infection, suggesting the potential of ATF3 as a therapeutic target for the treatment of herpesvirus-related diseases.

## Materials and methods

### Cell culture

The HEK293T, HEK293T.219, iSLK-RGB and iSLK-BAC16 cell lines were maintained in high-glucose Dulbecco’s modified Eagle’s medium (DMEM, HyClone), supplemented with 10% fetal bovine serum (FBS, Biological Industries) and 1% antibiotics (penicillin and streptomycin, Gibco). HEK293T.219, iSLK-RGB and iSLK-BAC16 cell lines needed respective selective agents (puromycin, 1.5 μg/ml; G418, 0.5 mg/ml; hygromycin, 0.5 mg/ml). KSHV-negative BJAB, KSHV-positive B lymphoma cell lines BJAB-KSHV and BCBL-1 were cultured in PRMI 1640 (HyClone) supplemented with 10% FBS and 1% antibiotics. All cells were cultured at 37 ˚C in a humidified environment supplemented with 5% CO_2_.

### Antibodies and reagents

The following primary antibodies were used: anti-ATF3 rabbit monoclonal antibody (Abcam, ab207434), anti-K8 mouse monoclonal antibody (Santa Cruz, SC-69797), anti-Mouse Control IgG antibody (Abclonal, AC011), anti-Rabbit Control IgG antibody (Abclonal, AC005), anti-GAPDH rabbit monoclonal antibody (Abclonal, A19056), anti-α-tubulin mouse monoclonal antibody (Sigma, T6199), anti-Flag antibody (Sigma, F7425 and F1804), anti-HA antibody (Sigma, H9658 and H6908), anti-LANA or RTA antibody were prepared in our laboratory. The secondary antibodies used in western blotting and immunofluorescence assays were HRP-conjugated anti-mouse or anti-rabbit IgG (Jackson ImmunoResearch Laboratories) and goat anti-mouse or anti-rabbit antibodies conjugated with Alexa Fluor 488, 555 and 647 (Thermo Fisher Scientific). The other used reagents and their sources were as follows: recombinant protein A agarose (Invitrogen, 15948–014), recombinant protein G agarose (Invitrogen, 15920–010), anti-Flag M2 affinity agarose (Sigma, A2220), 3 x Flag Peptide (Sigma, F4799), Lipofectamine 2000 (Thermo Fisher Scientific, 11668019), FuGENE HD Transfection Reagent (Promega, E2311), ATF3 agonist (MCE, HY-151923), doxycycline hyclate (Sigam, 324385), tetradecanoyl phorbol acetate (TPA, Sigma, P8139) and blasticidin (Sigma, 203350).

### Plasmids

The full-length fragment of ATF3 and K8 were amplified from the iSLK-RGB cDNA library, inserted into Flag-tagged pCDH and HA-tagged pCMV vectors. The ATF3 and K8 truncation and mutant plasmids were generated by using the pCMV-HA-RTA plasmid as a template following the manufacturer’s protocol of the Fast Site-Directed Mutagenesis Kit (TIANGEN). The luciferase reporter plasmids pGL3-Enhancer-pK8, pORF7, pORF26 and ATF3 were constructed by cloning the promoter regions of respective genes (-2000 to -1 bp) from the iSLK-RGB genomic library into the pGL3-Enhancer vector. The PCR primers used in this study were summarized in S4 Table.

### Mass spectrometry

Mass spectrometry was used to identify KSHV K8 protein complexes. HEK293T.219 cells were transfected with Flag-tagged K8 for 48 hours and iSLK-RGB-K8-Flag recombinant virus-infected cells were induced by doxycycline (1 μg/ml) for 72 hours. Cells were then washed two or three times with pre-cooled PBS and lysed in ice-cold radioimmunoprecipitation assay buffer (RIPA, Beyotime, P0013D) with 1 mM protease inhibitor (Beyotime, P1006) and protease inhibitor cocktail (Sigma, P8340). Following fully lysed, cells were rotated at 4 ˚C for 1 hours and then centrifuged at 13000 x rpm for 15 minutes at 4 ˚C. The cell lysates were then transferred to a new EP tube, and the cell debris were discarded. Flag M2 agarose beads were washed with RIPA buffer for three times and then resuspended by the same volume. Every 1 mL of cell lysates were added with 50 μL pre-mixed solution of M2 beads and then incubated with rotation at 4 ˚C overnight, followed by centrifugation at 3000 x rpm for 5 minutes at 4 ˚C to obtain the immunoprecipitants. After washed three times with 1 mL ice-cold RIPA buffer, the immunoprecipitants were resuspended in 50 μL 2 x loading buffer and were sent to company (APPLIED PROTEIN TECHNOLOGY) for mass spectrometric detection. Raw data of mass spectrometry analysis were provided by the company.

### Construction recombinant KSHV using the BAC system

Mutagenesis of RGB-BAC16 was performed as previously described [85]. Briefly, a linear DNA fragment was amplified via PCR using the pEpKan-S plasmid as a template, which contained a kanamycin cassette, an *I-SceI* restriction enzyme site, and duplicated flanking sequences derived from the regions of K8 corresponding coding sequences (approximately 40 bp copy). The PCR product was treated with *Dpn I* to remove the plasmid template, and the purified PCR fragments were electroporated into the GS1783 strain, following incubation in 1 mL LB medium without antibiotics at 32 ˚C for 1–2 hours. The recombinant clones were selected at 32 ˚C on LB plates containing chloramphenicol (12.5 μg/mL) and kanamycin (50 μg/mL). Positive clones were treated with 1% L-arabinose at 42 ˚C for 30 minutes, followed by incubated on LB plates containing 1% L-arabinose for secondary recombination at 32 ˚C. Clones from secondary recombination that were kanamycin sensitive and then were picked and confirmed by PCR and sequencing.

### Construction of the recombinant K8 related iSLK-RGB cell line

The recombinant RGB-BAC16 plasmids were extracted using the NucleoBond Xtra Midi Kit (Germany) and then iSLK-Puro cells were plated in 6-well plates. 4 μg plasmids were transfected into iSLK-Puro cells using 10 μL FuGENE HD Transfection Reagent (Promega). The medium of the iSLK-Puro cells was replaced with no FBS DMEM before transfection. At 2–3 hours after transfection, FBS was added to a final concentration at 10%. On the following day, the transfected cells were cultured in complete DMEM with a final concentration of hygromycin B (500 μg/mL) for selection. After two weeks of selection, hygromycin-resistant cells were trypsinized, pooled, and subcultured.

### Coimmunoprecipitation (co-IP) and Immunoblotting

Treated cells were lysed in RIPA buffer (Beyotime, P0013D) supplemented with protease inhibitor cocktail and 1mM PMSF on Rotational Incubator for 1 hour at 4˚C. Then the cells were centrifuged at 13,000 x rpm for 15 minutes at 4˚C to remove cell debris. Five to ten percent of the cell lysates were taken as the input, and the remainder was immunoprecipitated with affinity beads or the corresponding antibodies overnight at 4˚C. The immunoprecipitated were washed three times with RIPA buffer and boiled in SDS loading buffer at 100˚C for 10 minutes. For immunoblotting analysis, the treated protein samples were analyzed by SDS-PAGE and transferred to nitrocellulose membranes, followed by blocking with 5% skim milk powder in TBST buffer for 1 h at room temperature and probing with the indicated primary antibodies overnight at 4˚C. After hybridization with either goat anti-rabbit or goat anti-mouse secondary antibodies (diluted 1:5000) in TBST buffer, membranes were washed with TBST buffer three times (5–10 mins each) before visualization with ECL reagents (GE).

### Immunofluorescence assay

HEK293T or iSLK-BAC16 cells were plated onto coverslips in 6-well plates. At 48 hours after transfection or induction by doxycycline (1 μg/ml), cells were washed three times with PBS and fixed in 4% paraformaldehyde for 30 minutes. Cells were permeabilized with 0.2% Triton X-100 for 15 minutes and blocked for 30 minutes with 5% bovine serum albumin (BSA) in PBS, followed by incubation with the primary antibody overnight at 4 ˚C. Cells were washed three times with PBS and then incubated with responding secondary antibodies for 1 hour at room temperature. Cell nuclei were stained with DAPI (Beyotime, C1002) for 5 minutes. Finally, the coverslips were washed extensively and fixed onto slides. Slides were visualized and photographed by Zeiss confocal microscopy.

### RNA isolation and quantitative real-time (RT-qPCR)

Total RNA was isolated from cells using TRIzol reagent (Invitrogen) following the manufacturer’s instructions. Two micrograms of RNA was used for reverse transcription with gDNA Eraser reverse transcription kits (Toyobo). cDNA was used for quantification of the indicated mRNA on a QuantStudio 6 Flex Real-Time PCR System (Applied Biosystems) by using SYBR Green real-time PCR master mix kits (Toyobo) according to the manufacturer’s instructions. Dissociation curve analysis of products was conducted at the end of each PCR to detect and validate the specific amplification of PCR products. Transcript levels of each gene were normalized to the GAPDH level, and the 2 − ΔΔCT method was used to analyze gene expression in samples. To analyze viral genomic DNA level, intracellular viral genomic DNA and extracellular virion DNA were extracted from induced cells or the cell supernatants with the Genomic DNA Extraction Kit (TIANGEN). The KSHV genomic DNA copy numbers were quantified by RT-qPCR using primers K9. The standard curve was generated using serial dilutions of a PCDNA3.1-K9 plasmid. All the samples were tested in triplicate. The primers used in RT-qPCR were listed in S4 Table.

### Dual-luciferase reporter assay

HEK293T or HEK293T.219 cells were cultured in 12-well plates followed by transfecting with the indicated expression plasmids. At 48 hours post-transfection, cells were washed with PBS three times and lysed with 200 μL 1 x passive lysis buffer for 30 minutes at room temperature. 10 μL cell lysates were used to measure luminescence activity according to the manufacture’s instruction of dual-luciferase reporter assay system (Promega). The expressing Renilla luciferase plasmids pRL-TK were used to normalize firefly luciferase activity.

### RNA interference

Cells were transfected with negative control siRNA or siRNAs corresponding to the indicated genes (GenePharma Technology) using Lipofectamine 2000 according to the manufacturer’s instructions. At 24–48 hours post-transfection, the cells were harvested, and the efficiency of RNA interference was detected by immunoblotting analysis. All siRNA used in our experiments and their sequences are as follows:

Negative control siRNA, 5’-UUCUCCGAACGUGUCACGUTT-3’
siATF3-1, 5’-GAGGCGACGAGAAAGAAAUTT-3’
siATF3-2, 5’-GGAGUCCUCAUUGAAUCCUTT-3’
siK8-1, 5’- GCUCGCUGUUGUCAACCUACG-3’
siK8-2, 5’- GCGUGUCAUCGAAAGCAUACA-3’
siLANA-1, 5’- GAGAGGAAGUUGUAGGAAACG-3’
siLANA-2, 5’- GCAUUUGUGUCUAGUCCUACU-3’

### Establishment and identification of stable cell lines

ATF3-overexpressing lentiviruses were constructed based on the lentiviral vector pCDH-CMV-Flag-IRES-Blast. This ATF3-overexpressing lentiviral vector and empty vectors were packaged in HEK293T cells by co-transfection with the Δ8.9 packaging plasmid and a plasmid expressing vesicular stomatitis virus G protein (pVSV-G). At 48 hours post-transfection, the virus stock was collected and cleared by a 0.45 μm pore size filter. The ATF3 stably expressing iSLK-RGB cell lines were achieved by addition of the ATF3 stable expression lentiviral particles and centrifugation at 2500 x rpm for 2 hours. The medium was replaced by fresh DMEM. At 48 hours post-infection, the cells were screened with 25 μg/mL blasticidin, and immunoblotting was performed to determine the expression of ATF3.

### Chromatin immunoprecipitation (ChIP)

ChIP assay was performed using the BeyoChIP Enzymatic ChIP Assay kit (P2083S, Beyotime, China) according to the manufacturer’s instructions. Anti-ATF3 or anti-Flag antibodies were applied to immunoprecipitated protein from HEK293T.219 or HEK293T cell lysates with A/G magnetic beads conjugated to the anti-ATF3 or anti-Flag antibodies. DNA enrichment was assessed by qPCR and the primers used in ChIP assay were listed in S4 Table.

### Statistical analysis

GraphPad Prism software was used to perform the statistical analysis. Data were determined by the unpaired, two tailed Student’s t-tests and statistical significance was set as: ns, no significance, P-value >0.05; *, P-value<0.05; **, P-value<0.01; ***, P-value<0.001; ****, P-value<0.0001. Error bars represent as mean ± SD. Each experiment was carried out independently at least three times.

## Supporting information

S1 FigConstruction and analyses of a recombinant KSHV with a Flag tag fused to the C-terminal of K8 ORF.**(A)** Schematic diagram of RGB-BAC16 with a Flag tag fused to the C-terminal of K8 ORF. The kanamycin cassette with *I-SceI* recognition sequence, along with the Flag-tagged sequence, was generated by PCR with pEP-Kan plasmid as a template. This DNA fragment was cloned into RGB-BAC16 by homologous recombination. The kanamycin cassette was deleted by recombination with induction of *I-SceI* in bacteria by incubation with L-arabinose. **(B to D)** iSLK-RGB and iSLK-RGB-K8-Flag cells were induced by doxycycline (1 μg/ml) for 72 hours, and total cell lysates were subjected to immunoblotting with indicated antibodies (B); total RNA was extracted from cells to investigate the transcriptional level of indicated genes (C); intracellular viral genomic DNA (left panel) and extracellular virion DNA (right panel) were extracted from cell lysates or cell supernatants to quantify the KSHV genomic DNA copy numbers by qPCR analysis (D). For C and D, bars represent means ±SEM of triplicates from three independent experiments. The P values were calculated using Student’s t-test (two sides), and ns indicates no significance.(TIF)

S2 FigEndogenous ATF3 and K8 are colocalized in KSHV-positive iSLK-BAC16 cells.At 48 hours after the treatment of doxycycline (1 μg/ml), iSLK-BAC16 cells were fixed with 4% paraformaldehyde and labeled with mouse anti-K8 antibody and rabbit anti-ATF3 antibody, followed by incubation with goat anti-mouse IgG conjugated with Alexa Fluor 555 and goat anti-rabbit IgG conjugated with Alexa Fluor 647 to visualize the stained K8 and ATF3 proteins, respectively. Nuclei were labelled with DAPI. Cells were analyzed by Zeiss confocal microscopy and representative images with scale bars were shown.(TIF)

S3 Fig ATF3 expression is reduced in KSHV-positive cells. **(A)** Analysis of ATF3 expression profile in GSE2149 data. KSHV-negative and positive cell lines are indicated as IBL4, SM1, BCKN-1 and BC-1, BC-2, BC-5, respectively. **(B)** Analysis of ATF3 expression profile in GSE1880 data. KSHV-negative and positive cell lines are indicated as BJAB, DG75, Raji and BCBL-1, BC-1, BC-3, respectively. **(C and D)** ATF3 expression was reduced in KSHV-positive cell lines. Immunoblotting analysis was performed with the indicated antibodies in BJAB and BJAB-KSHV cells (C), as well as in MM and KMM cells (D). The protein level of ATF3 were quantified by densitometry and normalized to Tubulin level. For A and B, the P values were calculated using Student’s t-test (two sides). **P < 0.01.
(TIF)

S4 FigTreatment cells with ATF3 agonist impairs KSHV lytic replication.**(A to D)** iSLK-RGB cells were treated with or without ATF3 agonist (50 μM) for 24 hours, followed by doxycycline induction (1 μg/ml). Fluorescence microscopy images of eGFP-positive iSLK-RGB cells at the indicated time points were shown (A); total RNA was extracted from cells to investigate the transcriptional level of indicated genes (B); the extracellular virion DNA (C) and the intracellular viral genomic DNA (D) were extracted from cell supernatants or cell lysates to quantify the KSHV genomic DNA copy numbers by qPCR analysis. For B to D, cells were induced for another 72 hours and bars represent means ±SEM of triplicates from three independent experiments. The P values were calculated using Student’s t-test (two sides). *P < 0.05, **P < 0.01, ***P < 0.001, ****P < 0.0001.(TIF)

S5 FigEvaluation of the effects of two ATF3 truncations on KSHV lytic replication.**(A to C)** iSLK-RGB-CON, iSLK-RGB-ATF3-Full length, iSLK-RGB-ATF3-bZIP and iSLK-RGB-ATF3-ΔbZIP cells were induced by doxycycline (1 μg/ml) for 72 hours. Total RNA was extracted from cells to investigate the transcriptional level of indicated genes (A); the extracellular virion DNA (B) and the intracellular viral genomic DNA (C) were extracted from cell supernatants or cell lysates to quantify the KSHV genomic DNA copy numbers by qPCR analysis. For A to C, bars represent means ±SEM of triplicates from three independent experiments. The P values were calculated using Student’s t-test (two sides). **P < 0.01, ***P < 0.001, ****P < 0.0001, ns indicates no significance.(TIF)

S6 Fig Downregulation of endogenous ATF3 enhances KSHV lytic replication. **(A to D)** BCBL-1 cells were transfected with control siRNA and two ATF3-specific siRNAs. The knockdown efficiency was determined by immunoblotting (A); At 24 hours after transfection, cells were induced by TPA (20 ng/ml) for another 72 hours. The ATF3 and KSHV gene transcription level were analyzed by qPCR (B); the extracellular virion DNA (C) and the intracellular viral genomic DNA (D) were extracted from cell supernatants or cell lysates to quantify the KSHV genomic DNA copy numbers by qPCR analysis. For B to D, bars represent means ±SEM of triplicates from three independent experiments. The P values were calculated using Student’s t-test (two sides). ***P < 0.001, ****P < 0.0001.
(TIF)

S7 FigATF3 associates with K8 to repress *K8* promoter activity.**(A)** ATF3 has no effect on *K8* promoter activity in HEK293T cells. HEK293T cells were transfected with pK8 dual-reporter plasmid and increasing amounts of ATF3-expressing plasmids. At 48 hours after transfection, the cells were harvested and lysed in lysis buffer to detect luciferase activity. **(B)** ATF3-ΔbZIP has no effect on *K8* promoter activity in HEK293T cells, even if with K8 overexpression. HEK293T cells were transfected with pK8 dual-reporter plasmid, constant amounts of K8-expressing plasmids and full-length ATF3 or ATF3 with bZIP deletion truncation. At 48 hours after transfection, the cells were harvested and lysed in lysis buffer to detect luciferase activity. **(C)** ATF3-ΔbZIP has no effect on *K8* promoter activity in HEK293T.219 cells. HEK293T.219 cells were transfected with pK8 dual-reporter plasmid and full-length ATF3 or ATF3 with bZIP deletion truncation. At 6 hours after transfection, cells were induced by TPA (20 ng/ml) for another 48 hours and cells were harvested and lysed in lysis buffer to detect luciferase activity. For A to C, bars represent means ±SEM of triplicates from three independent experiments. The P values were calculated using Student’s t-test (two sides). ***P < 0.001, ns indicates no significance.(TIF)

S8 FigATF3 reducing K8 expression depends on its bZIP domain.**(A and B)** Effect of ATF3 on endogenous K8 expression. HEK293T.219 cells were transfected with control siRNA and two ATF3-specific siRNAs. At 24 hours after transfection, cells were induced by TPA (20 ng/ml) for another 48 hours and cells were harvested and analyzed by immunoblotting with the indicated antibodies (A); total RNA was extracted from cells to investigate the transcriptional level of K8 (B). **(C and D)** Effect of ATF3 on endogenous K8 expression. iSLK-RGB cells were transfected with control siRNA and two ATF3-specific siRNAs. At 6 hours after transfection, cells were induced by doxycycline (1 μg/ml) for another 72 hours and cells were harvested and analyzed by immunoblotting with the indicated antibodies (C); total RNA was extracted from cells to investigate the transcriptional level of K8 (D). For B and D, bars represent means ±SEM of triplicates from three independent experiments. The P values were calculated using Student’s t-test (two sides). **P < 0.01, ***P < 0.001, ****P < 0.0001.(TIF)

S9 Fig LANA decreases ATF3 expression by repressing its promoter. **(A)** Schematic diagram of *ATF3* promoter region (from -1200 bp to -720 bp). The putative LANA binding sites are underlined in red. **(B and C)** Effect of LANA on endogenous ATF3 expression. HEK293T.219 cells were transfected with control siRNA and two LANA-specific siRNAs. At 24 hours after transfection, cells were induced by TPA (20 ng/ml) for another 48 hours and cells were harvested and analyzed by immunoblotting with the indicated antibodies (B); total RNA was extracted from cells to investigate the transcriptional level of ATF3 (C). **(D)** Validation of LANA binding at the *ATF3* promoter. HEK293T cells were transfected with LANA-Flag for 48 hours and were subjected to ChIP assays using immunoglobulin G or the Flag antibody. The amounts of precipitated DNA were quantified by qPCR using primers amplifying the regions containing corresponding LANA binding sites. For C and D, bars represent means ±SEM of triplicates from three independent experiments. The P values were calculated using Student’s t-test (two sides). **P < 0.01, ***P < 0.001, ****P < 0.0001.
(TIF)

S10 FigATF3 associates with K8 to repress both *ORF7* and *ORF26* promoter activity.**(A)** Schematic diagram of *ORF7* promoter region (from -2000 bp to -1 bp). The ATF3 binding site (from -1511 bp to -1504 bp) is marked. **(B)** Schematic diagram of *ORF26* promoter region (from -2500 bp to -1 bp). The ATF3 binding site (from -2366 bp to -2359 bp) is marked. **(C and D)** With K8 overexpression, ATF3 suppresses *ORF7* (C) and *ORF26* (D) promoter activity in HEK293T cells. HEK293T cells were transfected with pORF7 or pORF26 dual-reporter plasmid and ATF3 or K8 expressing plasmids as indicated. At 48 hours after transfection, the cells were harvested and lysed in lysis buffer to detect luciferase activity. **(E)** iSLK-RGB-WT-CON, iSLK-RGB-WT-ATF3, iSLK-RGB-K8 Stop-CON and iSLK-RGB-K8 Stop-ATF3 cells were induced by doxycycline (1 μg/ml) for 72 hours and total RNA was extracted from cells to investigate the transcriptional level of indicated genes. For C to E, bars represent means ±SEM of triplicates from three independent experiments. The P values were calculated using Student’s t-test (two sides). *P < 0.05, **P < 0.01, ***P < 0.001, ns indicates no significance.(TIF)

S11 FigEvaluation of the effects of wildtype and its SUMO E3 ligase dead mutant K8 on ATF3 expression.**(A)** HEK293T cells were transfected with indicated plasmids for 48 hours and then cells were harvested to analyze the endogenous ATF3 expression by immunoblotting with the indicated antibodies. (B) iSLK-RGB-K8-Flag and iSLK-RGB-K8-L75A-Flag cells were induced with doxycycline (1 μg/ml) at different time points as indicated and cells were harvested to analyze the endogenous expression of ATF3 by immunoblotting with the indicated antibodies.(TIF)

S12 Fig ATF3 binding motifs are ubiquitous among human herpesviruses genomes. The chart shows the positions of ATF3 binding sites of human herpesviruses genomes and genes corresponding to these sites, which potentially regulated by ATF3. The sequences of human herpesviruses obtain from NCBI.
(TIF)

S1 Table Proteins interacted with KSHV K8 in both HEK293T.219 cells and iSLK-RGB-K8-Flag cells. (DOCX)

S2 Table Proteins interacted with KSHV K8 in iSLK-RGB-K8-Flag cells. (XLSX)

S3 Table Proteins interacted with KSHV K8 in HEK293T.219 cells. (XLSX)

S4 Table Primers used in this study. (XLSX)

S5 Table Raw data. (XLSX)
