# Supplementary material for: Transcriptional factor ATF3 impairs KSHV lytic replication by suppressing the expression of viral bZIP protein K8
Source: PLoS Pathog. 2026 May 11;22(5):e1014222. doi: 10.1371/journal.ppat.1014222 (PMC13178968; doi:10.1371/journal.ppat.1014222)
Supplement: S1 Table — (DOCX) [file ppat.1014222.s013.docx]

| **Uniport Number** | **Protein Function** |
| --- | --- |
| **P18847** | **Cyclic AMP-dependent transcription factor ATF-3 OS=Homo sapiens OX=9606 GN=ATF3 PE=1 SV=2** |
| Q9BW34 | EEF1D protein (Fragment) OS=Homo sapiens OX=9606 GN=EEF1D PE=2 SV=2 |
| Q96EB6 | NAD-dependent protein deacetylase sirtuin-1 OS=Homo sapiens OX=9606 GN=SIRT1 PE=1 SV=2 |
| I3L2C7 | Gem-associated protein 4 OS=Homo sapiens OX=9606 GN=GEMIN4 PE=1 SV=1 |
| E9PL71 | Elongation factor 1-delta (Fragment) OS=Homo sapiens OX=9606 GN=EEF1D PE=1 SV=1 |
| O15355 | Protein phosphatase 1G OS=Homo sapiens OX=9606 GN=PPM1G PE=1 SV=1 |
| Q6IAX2 | 60S ribosomal protein L21 OS=Homo sapiens OX=9606 GN=RPL21 PE=2 SV=1 |
| Q9C0C9 | (E3-independent) E2 ubiquitin-conjugating enzyme OS=Homo sapiens OX=9606 GN=UBE2O PE=1 SV=3 |
| C9J6P4 | Zinc finger CCCH-type antiviral protein 1 OS=Homo sapiens OX=9606 GN=ZC3HAV1 PE=1 SV=1 |
| Q05CW7 | NAT10 protein (Fragment) OS=Homo sapiens OX=9606 GN=NAT10 PE=2 SV=1 |
| Q14690 | Protein RRP5 homolog OS=Homo sapiens OX=9606 GN=PDCD11 PE=1 SV=3 |
| Q93008 | Probable ubiquitin carboxyl-terminal hydrolase FAF-X OS=Homo sapiens OX=9606 GN=USP9X PE=1 SV=4 |
| Q9Y4X5 | E3 ubiquitin-protein ligase ARIH1 OS=Homo sapiens OX=9606 GN=ARIH1 PE=1 SV=2 |
| D6RFN0 | COP9 signalosome complex subunit 4 OS=Homo sapiens OX=9606 GN=COPS4 PE=1 SV=1 |
| Q9Y2T2 | AP-3 complex subunit mu-1 OS=Homo sapiens OX=9606 GN=AP3M1 PE=1 SV=1 |
| O14617 | AP-3 complex subunit delta-1 OS=Homo sapiens OX=9606 GN=AP3D1 PE=1 SV=1 |
| O14974 | Protein phosphatase 1 regulatory subunit 12A OS=Homo sapiens OX=9606 GN=PPP1R12A PE=1 SV=1 |
| E7EM64 | COP9 signalosome complex subunit 6 OS=Homo sapiens OX=9606 GN=COPS6 PE=1 SV=1 |
| O00178 | GTP-binding protein 1 OS=Homo sapiens OX=9606 GN=GTPBP1 PE=1 SV=3 |
| P16402 | Histone H1.3 OS=Homo sapiens OX=9606 GN=H1-3 PE=1 SV=2 |
| Q8IWZ3 | Ankyrin repeat and KH domain-containing protein 1 OS=Homo sapiens OX=9606 GN=ANKHD1 PE=1 SV=1 |
| C5HU01 | Williams-Beuren syndrome chromosome region 14 protein 2 OS=Homo sapiens OX=9606 GN=MLXIPL PE=4 SV=1 |
| Q9UNS2 | COP9 signalosome complex subunit 3 OS=Homo sapiens OX=9606 GN=COPS3 PE=1 SV=3 |
| O00567 | Nucleolar protein 56 OS=Homo sapiens OX=9606 GN=NOP56 PE=1 SV=4 |
| Q7L5Y9 | E3 ubiquitin-protein transferase MAEA OS=Homo sapiens OX=9606 GN=MAEA PE=1 SV=1 |
| Q9H089 | Large subunit GTPase 1 homolog OS=Homo sapiens OX=9606 GN=LSG1 PE=1 SV=2 |
| A0A024R8L0 | Mitochondrial ribosomal protein S7, isoform CRA_a OS=Homo sapiens OX=9606 GN=MRPS7 PE=3 SV=1 |
| A0A087WYS3 | Protein kinase C-binding protein 1 OS=Homo sapiens OX=9606 GN=ZMYND8 PE=1 SV=1 |
| Q53FX9 | Mitochondrial ribosomal protein S11 isoform a variant (Fragment) OS=Homo sapiens OX=9606 PE=2 SV=1 |
| Q9BQ04 | RNA-binding protein 4B OS=Homo sapiens OX=9606 GN=RBM4B PE=1 SV=1 |
| Q8IX18 | Probable ATP-dependent RNA helicase DHX40 OS=Homo sapiens OX=9606 GN=DHX40 PE=1 SV=2 |
| J3KR49 | CLIP-associating protein 2 OS=Homo sapiens OX=9606 GN=CLASP2 PE=1 SV=1 |
| A0A0B4J1V8 | HCG2039996 OS=Homo sapiens OX=9606 GN=PPAN-P2RY11 PE=3 SV=1 |
| Q70CQ2 | Ubiquitin carboxyl-terminal hydrolase 34 OS=Homo sapiens OX=9606 GN=USP34 PE=1 SV=2 |
| Q15020 | Squamous cell carcinoma antigen recognized by T-cells 3 OS=Homo sapiens OX=9606 GN=SART3 PE=1 SV=1 |
| A0A024R4E5 | High density lipoprotein binding protein (Vigilin), isoform CRA_a OS=Homo sapiens OX=9606 GN=HDLBP PE=1 SV=1 |
| Q9HCG8 | Pre-mRNA-splicing factor CWC22 homolog OS=Homo sapiens OX=9606 GN=CWC22 PE=1 SV=3 |
| Q96F88 | Processing of 1, ribonuclease P/MRP subunit (S. cerevisiae) OS=Homo sapiens OX=9606 GN=POP1 PE=2 SV=1 |
| B2R4V2 | cDNA, FLJ92227, highly similar to Homo sapiens ribosomal protein L36a-like (RPL36AL), mRNA OS=Homo sapiens OX=9606 PE=2 SV=1 |
| Q12873 | Chromodomain-helicase-DNA-binding protein 3 OS=Homo sapiens OX=9606 GN=CHD3 PE=1 SV=3 |
| E9PKP7 | Nucleolar transcription factor 1 OS=Homo sapiens OX=9606 GN=UBTF PE=1 SV=1 |
| E9PGT6 | COP9 signalosome complex subunit 8 OS=Homo sapiens OX=9606 GN=COPS8 PE=1 SV=1 |
| Q96GQ7 | Probable ATP-dependent RNA helicase DDX27 OS=Homo sapiens OX=9606 GN=DDX27 PE=1 SV=2 |
| Q9UKD2 | mRNA turnover protein 4 homolog OS=Homo sapiens OX=9606 GN=MRTO4 PE=1 SV=2 |
| A0A0S2PZM4 | Bifunctional arginine demethylase and lysyl-hydroxylase JMJD6 OS=Homo sapiens OX=9606 GN=JMJD6 PE=4 SV=1 |
| Q9Y5J1 | U3 small nucleolar RNA-associated protein 18 homolog OS=Homo sapiens OX=9606 GN=UTP18 PE=1 SV=3 |
| Q96ME7 | Zinc finger protein 512 OS=Homo sapiens OX=9606 GN=ZNF512 PE=1 SV=2 |
| B2R8U0 | cDNA, FLJ94063, highly similar to Homo sapiens activating transcription factor 7 (ATF7), mRNA OS=Homo sapiens OX=9606 PE=2 SV=1 |
| Q13501 | Sequestosome-1 OS=Homo sapiens OX=9606 GN=SQSTM1 PE=1 SV=1 |
| D6RIC3 | Nucleolar protein 16 OS=Homo sapiens OX=9606 GN=NOP16 PE=1 SV=1 |
| Q9UKJ3 | G patch domain-containing protein 8 OS=Homo sapiens OX=9606 GN=GPATCH8 PE=1 SV=2 |
| Q59FP7 | DNA (cytosine-5)-methyltransferase 1 (Fragment) OS=Homo sapiens OX=9606 PE=2 SV=1 |
| P46013 | Proliferation marker protein Ki-67 OS=Homo sapiens OX=9606 GN=MKI67 PE=1 SV=2 |
| Q96AX1 | Vacuolar protein sorting-associated protein 33A OS=Homo sapiens OX=9606 GN=VPS33A PE=1 SV=1 |
| Q9Y4W2 | Ribosomal biogenesis protein LAS1L OS=Homo sapiens OX=9606 GN=LAS1L PE=1 SV=2 |
| B2RDG4 | cDNA, FLJ96597 OS=Homo sapiens OX=9606 PE=2 SV=1 |
| H3BND3 | Cleavage and polyadenylation specificity factor subunit 5 (Fragment) OS=Homo sapiens OX=9606 GN=NUDT21 PE=1 SV=8 |
| P49458 | Signal recognition particle 9 kDa protein OS=Homo sapiens OX=9606 GN=SRP9 PE=1 SV=2 |
| C9JJ19 | 28S ribosomal protein S34, mitochondrial OS=Homo sapiens OX=9606 GN=MRPS34 PE=1 SV=2 |
| Q5VWQ0 | Lysine-specific demethylase 9 OS=Homo sapiens OX=9606 GN=RSBN1 PE=1 SV=2 |
| A0A024R3V0 | DNA polymerase-transactivated protein 6, isoform CRA_a OS=Homo sapiens OX=9606 GN=DNAPTP6 PE=3 SV=1 |
| Q15334 | Lethal(2) giant larvae protein homolog 1 OS=Homo sapiens OX=9606 GN=LLGL1 PE=1 SV=3 |
| Q5VYS8 | Terminal uridylyltransferase 7 OS=Homo sapiens OX=9606 GN=TUT7 PE=1 SV=1 |
| Q9BXY0 | Protein MAK16 homolog OS=Homo sapiens OX=9606 GN=MAK16 PE=1 SV=2 |
| V9HWK0 | Signal recognition particle subunit SRP72 OS=Homo sapiens OX=9606 GN=HEL103 PE=2 SV=1 |
| Q8IV48 | 3'-5' exoribonuclease 1 OS=Homo sapiens OX=9606 GN=ERI1 PE=1 SV=3 |
| Q9H8Y5 | Ankyrin repeat and zinc finger domain-containing protein 1 OS=Homo sapiens OX=9606 GN=ANKZF1 PE=1 SV=1 |
| Q6PJJ2 | RRP1 protein (Fragment) OS=Homo sapiens OX=9606 GN=RRP1 PE=2 SV=2 |
| A0A286YFD0 | Methylenetetrahydrofolate reductase OS=Homo sapiens OX=9606 GN=MTHFR PE=1 SV=1 |
| Q9H8H2 | Probable ATP-dependent RNA helicase DDX31 OS=Homo sapiens OX=9606 GN=DDX31 PE=1 SV=2 |
| P49721 | Proteasome subunit beta type-2 OS=Homo sapiens OX=9606 GN=PSMB2 PE=1 SV=1 |
| Q8TDD1 | ATP-dependent RNA helicase DDX54 OS=Homo sapiens OX=9606 GN=DDX54 PE=1 SV=2 |
| H0YJZ6 | Endoribonuclease Dicer OS=Homo sapiens OX=9606 GN=DICER1 PE=1 SV=2 |
| Q9H4L4 | Sentrin-specific protease 3 OS=Homo sapiens OX=9606 GN=SENP3 PE=1 SV=2 |
| Q9H4L7 | SWI/SNF-related matrix-associated actin-dependent regulator of chromatin subfamily A containing DEAD/H box 1 OS=Homo sapiens OX=9606 GN=SMARCAD1 PE=1 SV=2 |
| Q96EL2 | 28S ribosomal protein S24, mitochondrial OS=Homo sapiens OX=9606 GN=MRPS24 PE=1 SV=1 |
| P42695 | Condensin-2 complex subunit D3 OS=Homo sapiens OX=9606 GN=NCAPD3 PE=1 SV=2 |
| B5MCF9 | Pescadillo homolog OS=Homo sapiens OX=9606 GN=PES1 PE=1 SV=1 |
| H0YMD0 | Annexin (Fragment) OS=Homo sapiens OX=9606 GN=ANXA2 PE=1 SV=1 |
| A0A2R8Y595 | Transcriptional repressor CTCF OS=Homo sapiens OX=9606 GN=CTCF PE=1 SV=1 |
| O94888 | UBX domain-containing protein 7 OS=Homo sapiens OX=9606 GN=UBXN7 PE=1 SV=2 |
| O60293 | Zinc finger C3H1 domain-containing protein OS=Homo sapiens OX=9606 GN=ZFC3H1 PE=1 SV=3 |
| A8KAN5 | cDNA FLJ75097, highly similar to Homo sapiens upstream binding protein 1 (LBP-1a) (UBP1), mRNA OS=Homo sapiens OX=9606 PE=2 SV=1 |
| M0R080 | DnaJ homolog subfamily B member 1 (Fragment) OS=Homo sapiens OX=9606 GN=DNAJB1 PE=1 SV=1 |
| Q12986 | Transcriptional repressor NF-X1 OS=Homo sapiens OX=9606 GN=NFX1 PE=1 SV=2 |
| P82914 | 28S ribosomal protein S15, mitochondrial OS=Homo sapiens OX=9606 GN=MRPS15 PE=1 SV=1 |
| B3KV02 | cDNA FLJ41015 fis, clone UTERU2018712, highly similar to COP9 signalosome complex subunit 7b OS=Homo sapiens OX=9606 PE=2 SV=1 |
| A0A024R8D4 | Mitochondrial ribosomal protein S2, isoform CRA_a OS=Homo sapiens OX=9606 GN=MRPS2 PE=3 SV=1 |
| Q9NYB0 | Telomeric repeat-binding factor 2-interacting protein 1 OS=Homo sapiens OX=9606 GN=TERF2IP PE=1 SV=1 |
| Q9BV38 | WD repeat-containing protein 18 OS=Homo sapiens OX=9606 GN=WDR18 PE=1 SV=2 |
| Q8IWR0 | Zinc finger CCCH domain-containing protein 7A OS=Homo sapiens OX=9606 GN=ZC3H7A PE=1 SV=1 |
| Q9HD72 | Krueppel-related zinc finger protein SBZF5 OS=Homo sapiens OX=9606 PE=2 SV=1 |
| A0A2R8Y566 | RAB11-binding protein RELCH OS=Homo sapiens OX=9606 GN=RELCH PE=1 SV=1 |
| Q9HC36 | rRNA methyltransferase 3, mitochondrial OS=Homo sapiens OX=9606 GN=MRM3 PE=1 SV=2 |
| P35250 | Replication factor C subunit 2 OS=Homo sapiens OX=9606 GN=RFC2 PE=1 SV=3 |
| Q6PCB5 | Lysine-specific demethylase RSBN1L OS=Homo sapiens OX=9606 GN=RSBN1L PE=1 SV=2 |
| Q9BXS6 | Nucleolar and spindle-associated protein 1 OS=Homo sapiens OX=9606 GN=NUSAP1 PE=1 SV=1 |
| B7ZBH1 | Eukaryotic translation initiation factor 6 (Fragment) OS=Homo sapiens OX=9606 GN=EIF6 PE=1 SV=1 |
| P07858 | Cathepsin B OS=Homo sapiens OX=9606 GN=CTSB PE=1 SV=3 |
| Q02790 | Peptidyl-prolyl cis-trans isomerase FKBP4 OS=Homo sapiens OX=9606 GN=FKBP4 PE=1 SV=3 |
| Q96QR8 | Transcriptional activator protein Pur-beta OS=Homo sapiens OX=9606 GN=PURB PE=1 SV=3 |
| B1APJ0 | cDNA FLJ57459, highly similar to Mitochondrial import inner membrane translocase subunit Tim23 OS=Homo sapiens OX=9606 PE=2 SV=1 |
| A0A0D9SF58 | Chromosome transmission fidelity protein 18 homolog OS=Homo sapiens OX=9606 GN=CHTF18 PE=1 SV=1 |
| Q96HR8 | H/ACA ribonucleoprotein complex non-core subunit NAF1 OS=Homo sapiens OX=9606 GN=NAF1 PE=1 SV=2 |
| P36957 | Dihydrolipoyllysine-residue succinyltransferase component of 2-oxoglutarate dehydrogenase complex, mitochondrial OS=Homo sapiens OX=9606 GN=DLST PE=1 SV=4 |
| Q14691 | DNA replication complex GINS protein PSF1 OS=Homo sapiens OX=9606 GN=GINS1 PE=1 SV=1 |
| O15047 | Histone-lysine N-methyltransferase SETD1A OS=Homo sapiens OX=9606 GN=SETD1A PE=1 SV=3 |
| Q7Z2W9 | 39S ribosomal protein L21, mitochondrial OS=Homo sapiens OX=9606 GN=MRPL21 PE=1 SV=2 |
| B4DVR4 | cDNA FLJ60912, highly similar to Vinexin OS=Homo sapiens OX=9606 PE=2 SV=1 |
| A0A0S2Z5V5 | Methyltransferase like 17 isoform 1 (Fragment) OS=Homo sapiens OX=9606 GN=METTL17 PE=2 SV=1 |
| Q9UNE7 | E3 ubiquitin-protein ligase CHIP OS=Homo sapiens OX=9606 GN=STUB1 PE=1 SV=2 |
| P0DPB6 | DNA-directed RNA polymerases I and III subunit RPAC2 OS=Homo sapiens OX=9606 GN=POLR1D PE=1 SV=1 |
| F5GX80 | Zinc finger CCHC domain-containing protein 8 (Fragment) OS=Homo sapiens OX=9606 GN=ZCCHC8 PE=1 SV=1 |
| B7Z9J8 | cDNA, FLJ78862, highly similar to Isocitrate dehydrogenase OS=Homo sapiens OX=9606 PE=2 SV=1 |
| Q8NB28 | Dynactin 3 (P22), isoform CRA_a OS=Homo sapiens OX=9606 GN=DCTN3 PE=2 SV=1 |
| Q6UX04 | Spliceosome-associated protein CWC27 homolog OS=Homo sapiens OX=9606 GN=CWC27 PE=1 SV=1 |
| A0A024RAL5 | Zinc finger, CCHC domain containing 9, isoform CRA_a OS=Homo sapiens OX=9606 GN=ZCCHC9 PE=4 SV=1 |
| A8K5H6 | Exonuclease 1 OS=Homo sapiens OX=9606 PE=2 SV=1 |
| B2R823 | RNA-binding protein PNO1 OS=Homo sapiens OX=9606 PE=2 SV=1 |
| Q5VV42 | Threonylcarbamoyladenosine tRNA methylthiotransferase OS=Homo sapiens OX=9606 GN=CDKAL1 PE=1 SV=1 |
| A0A024R4Z4 | HCG2039447, isoform CRA_d OS=Homo sapiens OX=9606 GN=hCG_2039447 PE=1 SV=1 |
| B7Z321 | Exocyst complex component Sec8 OS=Homo sapiens OX=9606 PE=2 SV=1 |
| O15381 | Nuclear valosin-containing protein-like OS=Homo sapiens OX=9606 GN=NVL PE=1 SV=1 |
| Q6P453 | Ubiquitin carboxyl-terminal hydrolase (Fragment) OS=Homo sapiens OX=9606 GN=USP11 PE=2 SV=1 |
| A0A024R9D2 | Metadherin, isoform CRA_a OS=Homo sapiens OX=9606 GN=MTDH PE=4 SV=1 |
| O60343 | TBC1 domain family member 4 OS=Homo sapiens OX=9606 GN=TBC1D4 PE=1 SV=2 |
| H7C5V3 | 28S ribosomal protein S28, mitochondrial (Fragment) OS=Homo sapiens OX=9606 GN=MRPS28 PE=1 SV=8 |
| Q53GZ2 | Polymerase (RNA) III (DNA directed) polypeptide E variant (Fragment) OS=Homo sapiens OX=9606 PE=2 SV=1 |
| Q8N1G2 | Cap-specific mRNA (nucleoside-2'-O-)-methyltransferase 1 OS=Homo sapiens OX=9606 GN=CMTR1 PE=1 SV=1 |
| B4DFG0 | Protein DEK OS=Homo sapiens OX=9606 GN=DEK PE=1 SV=1 |
| A0A0A0MTB8 | WD repeat-containing protein 36 OS=Homo sapiens OX=9606 GN=WDR36 PE=1 SV=1 |
| H7C1I7 | Zinc finger MYM-type protein 4 (Fragment) OS=Homo sapiens OX=9606 GN=ZMYM4 PE=1 SV=1 |
| A0A024R0H6 | RNA polymerase II-associated factor 1 homolog OS=Homo sapiens OX=9606 GN=PAF1 PE=3 SV=1 |
| B4DQK8 | Pyrroline-5-carboxylate reductase OS=Homo sapiens OX=9606 PE=2 SV=1 |
| Q5CZ91 | Methionine aminopeptidase OS=Homo sapiens OX=9606 GN=DKFZp781C0419 PE=3 SV=1 |
| A6ND22 | 28S ribosomal protein S16, mitochondrial OS=Homo sapiens OX=9606 GN=MRPS16 PE=1 SV=1 |
| Q5T1J5 | Putative coiled-coil-helix-coiled-coil-helix domain-containing protein CHCHD2P9, mitochondrial OS=Homo sapiens OX=9606 GN=CHCHD2P9 PE=5 SV=1 |
| O00458 | Interferon-related developmental regulator 1 OS=Homo sapiens OX=9606 GN=IFRD1 PE=1 SV=4 |
| Q9BY44 | Eukaryotic translation initiation factor 2A OS=Homo sapiens OX=9606 GN=EIF2A PE=1 SV=3 |
| Q13395 | Probable methyltransferase TARBP1 OS=Homo sapiens OX=9606 GN=TARBP1 PE=1 SV=1 |
| A0A024RE13 | Ribonuclease III, nuclear, isoform CRA_a OS=Homo sapiens OX=9606 GN=RNASEN PE=3 SV=1 |
| Q12899 | Tripartite motif-containing protein 26 OS=Homo sapiens OX=9606 GN=TRIM26 PE=1 SV=1 |
| Q9H2P0 | Activity-dependent neuroprotector homeobox protein OS=Homo sapiens OX=9606 GN=ADNP PE=1 SV=1 |
| Q9Y5T5 | Ubiquitin carboxyl-terminal hydrolase 16 OS=Homo sapiens OX=9606 GN=USP16 PE=1 SV=1 |
| Q9Y6D5 | Brefeldin A-inhibited guanine nucleotide-exchange protein 2 OS=Homo sapiens OX=9606 GN=ARFGEF2 PE=1 SV=3 |
| Q68D16 | [Tau protein] kinase (Fragment) OS=Homo sapiens OX=9606 GN=DKFZp686D0638 PE=2 SV=1 |
| P51530 | DNA replication ATP-dependent helicase/nuclease DNA2 OS=Homo sapiens OX=9606 GN=DNA2 PE=1 SV=3 |
| A0AVN2 | BRCA1 associated RING domain 1 OS=Homo sapiens OX=9606 GN=BARD1 PE=1 SV=1 |
| B2R960 | cDNA, FLJ94230, highly similar to Homo sapiens thioredoxin-like 1 (TXNL1), mRNA OS=Homo sapiens OX=9606 PE=2 SV=1 |
| C9JZB0 | Inositol polyphosphate 5-phosphatase K (Fragment) OS=Homo sapiens OX=9606 GN=INPP5K PE=1 SV=1 |
| Q9HCN4 | GPN-loop GTPase 1 OS=Homo sapiens OX=9606 GN=GPN1 PE=1 SV=1 |
| B1AXG1 | Non-specific serine/threonine protein kinase OS=Homo sapiens OX=9606 GN=RPS6KA3 PE=1 SV=2 |
| B2R9J4 | cDNA, FLJ94423, highly similar to Homo sapiens mitochondrial ribosomal protein L23 (MRPL23), nuclear gene encoding mitochondrial protein, mRNA OS=Homo sapiens OX=9606 PE=2 SV=1 |
| Q9BT22 | Chitobiosyldiphosphodolichol beta-mannosyltransferase OS=Homo sapiens OX=9606 GN=ALG1 PE=1 SV=2 |
| A0A024RBG3 | Mitochondrial ribosomal protein L42, isoform CRA_b OS=Homo sapiens OX=9606 GN=MRPL42 PE=3 SV=1 |
| Q5TZP7 | DNA-(apurinic or apyrimidinic site) endonuclease OS=Homo sapiens OX=9606 GN=APEX1 PE=2 SV=1 |
| H0Y3M2 | BUB3-interacting and GLEBS motif-containing protein ZNF207 (Fragment) OS=Homo sapiens OX=9606 GN=ZNF207 PE=1 SV=2 |
| H3BR95 | GATOR complex protein WDR59 (Fragment) OS=Homo sapiens OX=9606 GN=WDR59 PE=1 SV=1 |
| A0A0S2Z3W7 | Epididymis secretory sperm binding protein (Fragment) OS=Homo sapiens OX=9606 GN=ITPA PE=2 SV=1 |
| Q12797 | Aspartyl/asparaginyl beta-hydroxylase OS=Homo sapiens OX=9606 GN=ASPH PE=1 SV=3 |
| C9JBY7 | 28S ribosomal protein S33, mitochondrial OS=Homo sapiens OX=9606 GN=MRPS33 PE=1 SV=1 |
| Q53X93 | CREB1 protein (Fragment) OS=Homo sapiens OX=9606 GN=CREB1 PE=2 SV=1 |
| Q9BRU9 | rRNA-processing protein UTP23 homolog OS=Homo sapiens OX=9606 GN=UTP23 PE=1 SV=2 |
| Q9H9L3 | Interferon-stimulated 20 kDa exonuclease-like 2 OS=Homo sapiens OX=9606 GN=ISG20L2 PE=1 SV=1 |
| Q53FN7 | BZW1 protein variant (Fragment) OS=Homo sapiens OX=9606 PE=2 SV=1 |
| B4DDK2 | cDNA FLJ57044, highly similar to Homo sapiens fetal Alzheimer antigen (FALZ), transcript variant 1, mRNA OS=Homo sapiens OX=9606 PE=2 SV=1 |
| Q712K3 | Ubiquitin-conjugating enzyme E2 R2 OS=Homo sapiens OX=9606 GN=UBE2R2 PE=1 SV=1 |
| Q8WVY7 | Ubiquitin-like domain-containing CTD phosphatase 1 OS=Homo sapiens OX=9606 GN=UBLCP1 PE=1 SV=2 |
| A8K2S7 | cDNA FLJ77865 OS=Homo sapiens OX=9606 PE=2 SV=1 |
| J3KNN7 | BRCA1-associated protein OS=Homo sapiens OX=9606 GN=BRAP PE=1 SV=1 |
| A0A669KB29 | CXXC-type zinc finger protein 1 OS=Homo sapiens OX=9606 GN=CXXC1 PE=1 SV=1 |
| P57081 | tRNA (guanine-N(7)-)-methyltransferase non-catalytic subunit WDR4 OS=Homo sapiens OX=9606 GN=WDR4 PE=1 SV=2 |
| Q9H9B1 | Histone-lysine N-methyltransferase EHMT1 OS=Homo sapiens OX=9606 GN=EHMT1 PE=1 SV=4 |
| Q13895 | Bystin OS=Homo sapiens OX=9606 GN=BYSL PE=1 SV=3 |
| B4DST5 | cDNA FLJ58078, highly similar to Tyrosine-protein phosphatase non-receptortype 23 OS=Homo sapiens OX=9606 PE=2 SV=1 |
| Q59HH0 | Retinoblastoma-associated protein variant (Fragment) OS=Homo sapiens OX=9606 PE=2 SV=1 |
| Q92538 | Golgi-specific brefeldin A-resistance guanine nucleotide exchange factor 1 OS=Homo sapiens OX=9606 GN=GBF1 PE=1 SV=2 |
| C9JA93 | TBC1 domain family member 15 (Fragment) OS=Homo sapiens OX=9606 GN=TBC1D15 PE=1 SV=1 |
| Q13451 | Peptidyl-prolyl cis-trans isomerase FKBP5 OS=Homo sapiens OX=9606 GN=FKBP5 PE=1 SV=2 |
| B2RTU6 | HCG1981012, isoform CRA_b OS=Homo sapiens OX=9606 GN=RAPGEF6 PE=2 SV=1 |
| Q59FX3 | Neurofibromin variant (Fragment) OS=Homo sapiens OX=9606 PE=2 SV=1 |
| H6QX63 | Hepatocellular carcinoma related protein 2 OS=Homo sapiens OX=9606 PE=2 SV=1 |
| O95707 | Ribonuclease P protein subunit p29 OS=Homo sapiens OX=9606 GN=POP4 PE=1 SV=2 |
| Q14126 | Desmoglein-2 OS=Homo sapiens OX=9606 GN=DSG2 PE=1 SV=2 |
| Q13042 | Cell division cycle protein 16 homolog OS=Homo sapiens OX=9606 GN=CDC16 PE=1 SV=2 |
| B4DMM7 | cDNA FLJ59722, highly similar to Retinoblastoma-binding protein 5 OS=Homo sapiens OX=9606 PE=2 SV=1 |
| Q8WWA6 | ZNF277 protein OS=Homo sapiens OX=9606 GN=ZNF277 PE=1 SV=1 |
| Q9NQC8 | Intraflagellar transport protein 46 homolog OS=Homo sapiens OX=9606 GN=IFT46 PE=1 SV=1 |
| A0A087WYR0 | Signal recognition particle 19 kDa protein OS=Homo sapiens OX=9606 GN=SRP19 PE=1 SV=1 |
| Q9H6F5 | Coiled-coil domain-containing protein 86 OS=Homo sapiens OX=9606 GN=CCDC86 PE=1 SV=1 |
| Q59E94 | Peroxisomal D3,D2-enoyl-CoA isomerase isoform 1 variant (Fragment) OS=Homo sapiens OX=9606 PE=2 SV=1 |
| F8W9B8 | Exocyst complex component 5 OS=Homo sapiens OX=9606 GN=EXOC5 PE=1 SV=1 |
| A0A024R120 | Transcription factor CP2, isoform CRA_a OS=Homo sapiens OX=9606 GN=TFCP2 PE=3 SV=1 |
| A0A024R7G7 | Solute carrier family 35, member E1, isoform CRA_c OS=Homo sapiens OX=9606 GN=SLC35E1 PE=4 SV=1 |
| O95801 | Tetratricopeptide repeat protein 4 OS=Homo sapiens OX=9606 GN=TTC4 PE=1 SV=3 |
| Q92541 | RNA polymerase-associated protein RTF1 homolog OS=Homo sapiens OX=9606 GN=RTF1 PE=1 SV=4 |
| Q9NTK5 | Obg-like ATPase 1 OS=Homo sapiens OX=9606 GN=OLA1 PE=1 SV=2 |
| A0A024RBX2 | Family with sequence similarity 51, member A1, isoform CRA_a OS=Homo sapiens OX=9606 GN=FAM51A1 PE=4 SV=1 |
| Q8WVC0 | RNA polymerase-associated protein LEO1 OS=Homo sapiens OX=9606 GN=LEO1 PE=1 SV=1 |
| Q2M389 | WASH complex subunit 4 OS=Homo sapiens OX=9606 GN=WASHC4 PE=1 SV=2 |
| Q14192 | Four and a half LIM domains protein 2 OS=Homo sapiens OX=9606 GN=FHL2 PE=1 SV=3 |
| B4DTN6 | cDNA FLJ60152, highly similar to Zinc finger protein 295 OS=Homo sapiens OX=9606 PE=2 SV=1 |
| Q8WXC6 | COP9 signalosome complex subunit 9 OS=Homo sapiens OX=9606 GN=COPS9 PE=1 SV=3 |
| O94822 | E3 ubiquitin-protein ligase listerin OS=Homo sapiens OX=9606 GN=LTN1 PE=1 SV=6 |
| A0A024R6F2 | Zinc finger CCCH domain-containing protein 14 OS=Homo sapiens OX=9606 GN=FLJ11806 PE=3 SV=1 |
| Q6RFH5 | WD repeat-containing protein 74 OS=Homo sapiens OX=9606 GN=WDR74 PE=1 SV=1 |
| Q9UNN5 | FAS-associated factor 1 OS=Homo sapiens OX=9606 GN=FAF1 PE=1 SV=2 |
| Q9NW82 | WD repeat-containing protein 70 OS=Homo sapiens OX=9606 GN=WDR70 PE=1 SV=1 |
| A0A1B0GVD3 | Protein lin-28 homolog B OS=Homo sapiens OX=9606 GN=LIN28B PE=1 SV=1 |
| A0A3B3IUC4 | Alpha-galactosidase OS=Homo sapiens OX=9606 GN=GLA PE=1 SV=1 |
| F8WAY1 | Lariat debranching enzyme OS=Homo sapiens OX=9606 GN=DBR1 PE=1 SV=1 |
